# A ferroptosis-related gene signature associated with immune landscape and therapeutic response in osteosarcoma

**DOI:** 10.3389/fonc.2022.1024915

**Published:** 2022-11-11

**Authors:** Xinxing Wang, Guang Xia, Shilang Xiao, Song Wu, Lina Zhang, Junjie Huang, Wenxiu Zhang, Xu Cao

**Affiliations:** ^1^ Department of Orthopaedics, The Third Xiangya Hospital, Central South University, Changsha, China; ^2^ Department of Gastroenterology, The Third Xiangya Hospital, Central South University, Changsha, China

**Keywords:** ferroptosis, osteosarcoma, immune microenvironment, prognostic risk model, sing-cell sequencing

## Abstract

**Background:**

The role of ferroptosis in tumor progression and immune microenvironment is extensively investigated. However, the potential value of ferroptosis regulators in predicting prognosis and therapeutic strategies for osteosarcoma (OS) patients remains to be elucidated.

**Methods:**

Here, we extracted transcriptomic and survival data from Therapeutically Applicable Research to Generate Effective Treatments (TARGET) and Gene Expression Omnibus (GEO) to investigate the expression and prognostic value of ferroptosis regulators in OS patients. After comprehensive analyses, including Gene set variation analysis (GSVA), single-sample gene-set enrichment analysis (ssGSEA), Estimated Stromal and Immune cells in Malignant Tumor tissues using Expression (ESTIMATE), single-cell RNA sequencing, and biological experiments, our constructed 8-ferroptosis-regulators prognostic signature effectively predicted the immune landscape, prognosis, and chemoradiotherapy strategies for OS patients.

**Results:**

We constructed an 8-ferroptosis-regulators signature that could predict the survival outcome of OS. The signature algorithm scored samples, and high-scoring patients were more prone to worse prognoses. The tumor immune landscape suggested the positive relevance between risk score and immunosuppression. Interfering HILPDA and MUC1 expression would inhibit tumor cell proliferation and migration, and MUC1 might improve the ferroptosis resistance of OS cells. Moreover, we predicted chemoradiotherapy strategies of cancer patients following ferroptosis-risk-score groups.

**Conclusion:**

Dysregulated ferroptosis gene expression can affect OS progression by affecting the tumor immune landscape and ferroptosis resistance. Our risk model can predict OS survival outcomes, and we propose that HILPDA and MUC1 are potential targets for cancer therapy.

## Introduction

Osteosarcoma (OS), the most common bone tumor, is a highly aggressive malignancy that frequently occurs in childhood and adolescence and has a worldwide annual incidence rate of 1~3 cases per million (1). OS originates from primitive mesenchymal cells in bone and rarely in soft tissue and progresses to pulmonary metastasis, whose subsequent relapse remains the primary cause of OS-related death (2). The current treatment strategy for OS patients includes neoadjuvant chemotherapy combined with surgical removal of the primary lesions and evidenced metastatic lesions, followed by additional adjuvant chemotherapy (3). Compared with management regimens before 1970, multiagent chemotherapy has considerably improved the long-term survival of localized OS patients from 20% to 70%. However, metastatic and recurrent OS patients still have a significantly low survival rate (4). Unfortunately, since the mid-1970s, little progress has been made in improving standard management strategies and increasing the survival rate of OS patients (3). The therapeutic outcome of OS is significantly impacted by intrinsic cellular heterogeneity and complex immunogenic mechanisms (5). Immune checkpoint inhibitors have made breakthroughs in the immunotherapy of various cancers (6, 7), whereas the therapeutical effect of targeting TILs and PD-L1 in managing OS is inconsistent (8–11). These suggest that OS might have a complex immune status that helps cancer cells evade the immune surveillance-mediated cell death. Therefore, identifying novel effective immune therapeutic targets to benefit treatment for OS is needed. Recently, three newly identified types of cell death, including ferroptosis, necroptosis, and pyroptosis, have been suggested to have crosstalk with antitumor immunity (12). As a research hotspot, ferroptosis was involved in multiple antitumor mechanisms. However, the relationship between ferroptosis and OS immune microenvironment remains to be elucidated.

Ferroptosis, distinguished from traditional cell death-like apoptosis, cell autophagy, or necroptosis, is a novel programmed cell death characterized by iron-dependent lipid peroxidation (13). Previous studies have suggested that ferroptosis regulators, including GPX4 (14), FANCD2 (15), P53 (16), and HSPB1 (17), are related to oncogenesis and progression. Increasing evidence has identified the pivotal role of ferroptosis in tumor therapies (18–20), in addition to the sensitivity of various tumors to ferroptosis, such as ovarian cancer (21), hepatocellular carcinoma (22), and adrenocortical carcinomas (23). Notably, the anti-tumorigenesis effect of ferroptosis is likely propelled by the immune system. Wang et al. (24) reported that CD8+ T cells released interferon-gamma (IFNγ) could induce ferroptosis activity in cancer cells. On the contrary, ferroptosis-induced regulatory factors and the release of micromolecules may contribute to immunosuppression and tumor growth (25). Hence, the regulatory network between immune responses and ferroptosis as it relates to tumor immunotherapy remains unclear. In attempts to address this gap, several studies have suggested a correlation between ferroptosis regulators and antitumor drug sensitivity in treating OS (26–29).

In this study, we collected data from Therapeutically Applicable Research to Generate Effective Treatments (TARGET) and Gene Expression Omnibus (GEO) to investigate the expression and prognostic value of ferroptosis regulators in OS patients. Risk signatures were constructed based on selected ferroptosis genes to evaluate the prognostic value of ferroptosis in risk stratification. Single-cell sequencing analysis was performed to explore the interaction between ferroptosis regulators and the immune microenvironment. Additionally, we investigated the predictive value of ferroptosis signature in anticancer chemotherapy. We further verified the cancer promotion function of pivotal genes HILPDA and MUC1 and revealed the probable association between them and ferroptosis. Therefore, this study aimed to comprehensively assess the effect of ferroptosis regulators on the immune microenvironment, prognosis, and therapeutic efficacy in OS.

## Materials and methods

This study protocol was approved by the institutional review board (IRB) of the Third Xiangya Hospital, Central South University (No: 2020-S221). All experiments involving human tissues were performed based on guidelines approved by the IRB. Each sample was processed only after receiving a signed informed consent form.

### Data collection

Expression array profiling of 9 normal cell lines (5 normal osteoblast cells and 4 normal bone cells) and 103 patient-derived OS cell lines were extracted from GSE42352 (30) and GSE36001 on the GEO (https://www.ncbi.nlm.nih.gov/geo/). The batch effect was eliminated using the “removeBatchEffect” function in the R package “limma.” Expression heatmaps were visualized with the “pheatmap” R package, while boxplot was constructed using the “ggpubr” R package. TARGET-OS RNA-seq data of 84 OS patients with available clinical characteristics extracted from the UCSC Xena website (https://xenabrowser.net/) were analyzed as the training cohort (**Table S1**). Furthermore, 53 OS samples extracted from GSE21257 (31) in the GEO database were validation cohorts (**Table S2**). In each cohort, we used the following criteria to exclude unqualified samples: (a) follow-up time < 1 month; (b) lack of survival data; (c) histopathological type is not OS. These count matrixes were standardized using the “DEseq2” package. Single-cell RNA sequencing datasets containing two primary OS lesions, “BC21” and “BC22”, two metastatic OS lesions “BC10” and “BC17”, and two recurrent OS lesions “BC11” and “BC20” were collected from GSE152048 (32) in GEO database. Ferroptosis regulators, including 108 driver genes and 69 suppressor genes, were obtained from the FerrDb website (**Table S3**) (http://www.zhounan.org/ferrdb) (33).

### Non-negative matrix factorization clustering for ferroptosis regulators

One hundred seventy-three ferroptosis-related genes were extracted and analyzed in the TARGET-OS training cohort. Candidate regulators with a high median absolute deviation (MAD > 0.5) value across the OS patients were selected for subsequent NMF clustering analysis. Unsupervised NMF clustering was performed using the “NMF” R package based on the 132 candidate genes (34). When the coexistence correlation coefficient k = 2, we observed the clearest boundary and most appropriate consistency; thus, 84 patients were clustered into two subclusters. In addition, principal component analysis (PCA) was used to validate the subcluster distribution with the expression of candidate ferroptosis regulators.

### Gene set variation analysis and functional annotation

To explore the difference between ferroptosis-related subclusters in biological processes, we conducted a GSVA enrichment analysis using the “GSVA” R package (35). Two gene sets, “c2.cp.kegg.v7.4.symbols” and “c5.go.bp.v7.4.symbols” were obtained from MSigDB database for performing GSVA enrichment. Moreover, Gene Ontology (GO) term enrichment, Kyoto Encyclopedia of Genes and Genomes (KEGG) pathway analysis, and annotation were also conducted with “clusterProfiler” and “org.Hs.eg.db” R packages. Finally, histograms were developed with the “ggplot2” R package.

### Assessment of tumor microenvironment cell infiltration

We conducted a single-sample gene-set enrichment analysis (ssGSEA) algorithm to assess the expression abundance of 28 specific infiltrating immune cell types in the OS TME. Marker gene sets for these TME infiltrating immune cells were collected from previous studies, covering multiple immune cell types, including activated B cell, CD8+ T cell, macrophage, natural killer T cell, and others (36, 37). Estimated Stromal and Immune cells in Malignant Tumor tissues using Expression (ESTIMATE) analysis was performed using the “estimate” R package to evaluate the infiltration of stromal cells and immune cells. The ESTIMATE score based on stromal and immune scores was used to evaluate tumor purity (38), and Scatter diagrams were developed using the “ggplot2” R package.

### Construction of ferroptosis risk signature

Based on the 132 ferroptosis regulators for NMF clustering, we identified 22 independent prognosis-related genes with univariate Cox regression analysis (*P* < 0.05). Then, the least absolute shrinkage and selection operator (LASSO) algorithm filtered out 11 ferroptosis regulators that met the minimum lambda value. Finally, stepwise multivariate Cox regression analysis confirmed 8 genes with optimal collinearity, and a risk signature was constructed. A risk score of each OS patient in the TARGET training cohort and GEO validation cohort was calculated with the following algorithm:

Risk score = 0.705×ATF4 + 0.503×ATM + 0.616×HILPDA + 0.323×MUC1 + 0.417×CBS + 0.238×MT1G + (-0.969)×ARNTL + (-0.553)×PML.

Hazard ratios (HRs) were used to distinguish protective (HR < 1) and risky elements (HR > 1). Forest plots were developed using the “ggplot2” R package.

### Single-cell RNA sequencing analysis

scRNA-seq analysis was conducted as previously described (39, 40). All single-cell expression matrixes of primary, metastatic, and occurrent OS patients from GSE152048 were processed by the “Seurat” R package. Firstly, “NormalizedData” was applied to normalize these expression data, then we performed “FindVariableFeatures” to identify the 1,000 most variable genes. After PCA with “RunPCA,” we conducted a K-nearest neighbor graph *via* “FindNeighbors,” while cells were combined with the “FindClusters” function. Subsequently, Uniform Manifold Approximation and Projection for Dimension Reduction (UMAP) (41) was used for visualization. Moreover, we performed a “Single R” R package to annotate cells when feature genes for all concerned cell categories were obtained from reported studies (32). Then, the “FindMarkers” function was performed to find differentially expressed genes for identified risk clusters.

### Immunohistochemistry

Five pairs of formalin-fixed paraffin-embedded OS tissue and para-carcinoma tissue blocks (all post-chemotherapy) from 5 patients with OS were made into 5 µm paraffin sections. IHC was performed following the Mouse/rabbit enhanced polymer method detection system (ZSGB-BIO, PV-9000, China). The slides were deparaffinized and rehydrated using xylene and gradient-concentration ethyl alcohol, followed by antigen retrieval with sodium citrate at 95°C. At room temperature, the slides were blocked using an endogenous peroxidase blocker for 10 min. Samples were incubated with primary antibodies against HILPDA (Proteintech, China) and MUC1 (Proteintech, China) overnight at 4°C, reaction enhancer for 20 min at 37°C, and enhanced enzyme-conjugated sheep anti-mouse/rabbit IgG polymer for 20 min at 37°C. Then the slides were stained with 3, 30-diaminobenzidine tetrahydrochloride (DAB) and counterstained with hematoxylin. Images were captured with a magnification of 20x.

### Cells culture

Two osteosarcoma cell lines (U2OS and MNNG/HOS) were kindly provided by Procell Life Science & Technology Co., Ltd. U2OS and MNNG/HOS were correspondingly cultured in McCoy’s 5A (Procell, China), and MEM (Procell, China), both supplemented with 10% fetal bovine serum (Gibco, USA) and 1% penicillin-streptomycin solution (Biosharp, China) at 37°C with saturated humidity and 5% CO2. The average time of culture medium exchange was 24-48h. The cells were digested with trypsin-EDTA (Gibco, USA) and passaged when cell adhesion exceeded 80% confluency.

### Small interfering RNA transfection

Human HILPDA siRNA (si-HILPDA), MUC1 siRNA (si-MUC1), and their nonspecific control siRNA (si-NC) were synthesized by JTSBio (Wuhan, China). The siRNAs were transfected into cells using jetPRIME transfection reagent (Polyplus, France) following the manufacturer’s protocol. The siRNAs sequences were listed in **Table S4**. RNA extraction and cell proliferation assay were performed 48h after transfection.

### Western blot

A mixture of RIPA (Beyotime, China) and a final concentration of 1mM PMSF (Beyotime, China) was used to lyse cells for protein extraction. Loading Buffer (Biosharp, China) was added to the protein supernatant, and then the sample was boiled to denature the protein. Then proteins were separated using SDS–PAGE gel (Biosharp, China), transferred to PVDF membranes (Millipore, USA), and blocked in 5% skimmed milk for 1​h. Then membranes were incubated overnight at 4​°C with primary antibodies, including HILPDA (Proteintech, China), MUC1 (Proteintech, China), ASCL4 (Affinity, China), GPX4 (Affinity, China), xCT (Affinity, China) and GAPDH. The membranes were incubated with fluorophore-conjugated secondary antibody (LI-COR Corp, NE) the following day. Protein bands were captured with an enhanced LI-COR Odyssey infrared imaging system (LI-COR Corp, NE), and the protein levels were normalized to the GAPDH levels.

### Real-time quantitative polymerase chain reaction

RT-qPCR primers are listed in **Table S4**. Total RNA from cultured cells was extracted using Rnafast200 (Fastagen, Japan), and cDNA was synthesized using HiScript II Q RT SuperMix for qPCR (Vazyme, China). ChamQ Universal SYBR qPCR Master Mix (Vazyme, China) was used to conduct RT-qPCR based on the manufacturer’s protocol. All steps for RT-qPCR reaction were conducted as follows: initial denaturation at 95°C for 30s, one cycle; denaturation at 95°C for 10s, 40 cycles; dissolution curve at 95°C for 15s, 60°C for 60s, 95°C for 15s, one cycle. Gene expression levels were normalized to those of GAPDH and calculated using lg2–△△Ct method.

### EdU incorporation assay

Proliferating OS cells were identified using the Click-iT Plus EdU Alexa Fluor 488 Imaging Kit (Invitrogen, USA), and cell nuclei were stained using Hoechst (Invitrogen, USA). Image Pro-Plus version 6.0 (Media Cybernetics, USA) was applied to calculate the counts and percentage of EdU-positive cells.

### Cell migration assay

OS cell migration was assayed using a Transwell chamber (Corning, USA) with polycarbonic membranes (6.5 mm in diameter and 8 μm pore size). Cells in a serum-free medium were added into the upper chamber at the density of 5 × 10^5^ cells/ml (200 μl/well), and an OS-conditioned medium with 10% FBS was added to the lower chamber. After incubating for 48h at 37°C, non-migrated cells on the membrane were removed with a cotton swab. Cells that penetrated to the lower surface were stained with 0.1% crystal violet. Then cells in five random fields per well were counted under 200×magnification as n=1 for the assay in triplicate.

### Cell viability detection

The cells were seeded into 96-well plates at a density of 5,000 cells/well with specific-concentration RSL3 (Selleck, China). After 24h, 1/10 volume of CCK-8 reagent (Proteintech, USA) was added to the wells, and the absorbance value was detected at 450nm after 1h incubation at 37°C. The experiment was repeated three times.

### Lipid reactive oxygen species detection

BODIPY 581/591 C11(Invitrogen, D3861, USA) with a final concentration of 2μM was used to detect intracellular and lipid cell membrane ROS. After incubation for 30min at dark 37°C, cells were digested with trypsin and resuspended by PBS to prepare a 300 μl cell suspension to determine lipid oxidation by Flow Cytometry. The fluorescence intensity of the FITC channel was measured by BD FACS Canto II (BODIPY 581/591 C11 at 590 nm in the non-oxidized state and 510 nm in the oxidized state). At least 10,000 cells were analyzed per sample, and data were analyzed using FlwoJo V10.

### Potential therapeutic prediction value of ferroptosis signature

We extracted expression matrix and drug response data of over 1,000 cancer cell lines from the Genomics of Drug Sensitivity in Cancer (GDSC, http://www.cancerrxgene.org/) database (42). Afterward, each cell line’s risk scores were calculated by conducting a ferroptosis signature. Then, we performed the Spearman method to evaluate the correlation (Cor) between risk scores and half-maximal inhibitory concentration (IC50) of each cell line. | Cor | > 0.2 and *P* < 0.05 were considered statistically significant.

### Statistical analysis

All bioinformatics statistical analyses and visualization were performed using R version 4.0.3 (https://www.r-project.org/), and the R script was listed in **Supplementary “R_script”**. Kaplan–Meier and log-rank analysis using “survival” and “survminer” packages were applied to evaluate the survival comparison. Receiver operating characteristic (ROC) and the area under the curve (AUC) were conducted with the “Time ROC” R package. Spearman correlation analysis was applied to evaluate correlations among continuous variables. Wilcoxon and One-way Anova tests were used to compare the difference among groups. A Chi-square test was used to identify the significance of ferroptosis DEGs (differentially expressed genes) among all detected genes. Values in cell experiment are mean ± SD unless otherwise noted and analyzed using Graphpad Prism version 8.0.2.263. Furthermore, the Benjamini-Hochberg method was utilized to adjust p values in functional annotation. *P.adjust* < 0.05 was considered statistically significant.

## Results

### Expression of ferroptosis genes was disordered in OS cells

**Figure 1:**
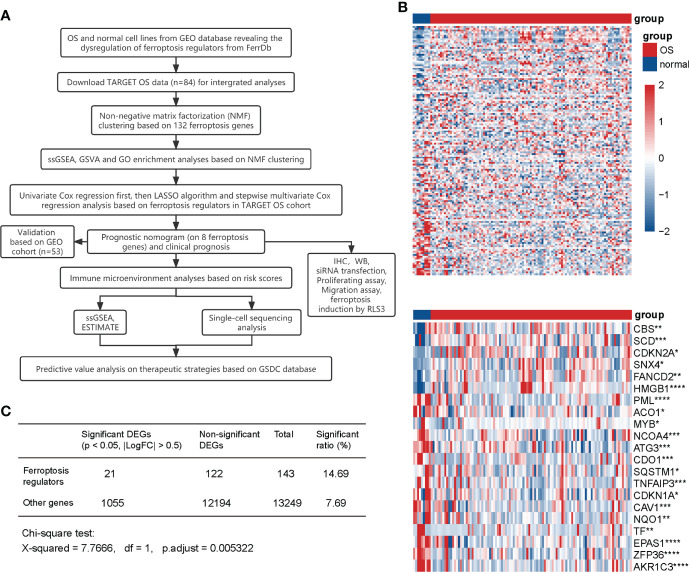
Expression of ferroptosis regulators in normal and OS cell lines. **(A)** Flow diagram of the study. **(B)** Heatmaps of the expression of 143 detected ferroptosis regulators (up) and 21 significant DEGs (|LogFC| > 0.5, *P <*0.05) (down). Red represents high expression level and blue represents low expression. The darker the color, the greater the significance. **(C)** Chi-square test for the significance of ferroptosis DEGs. * *P* < 0.05; ** *P* < 0.01; *** *P* < 0.001; **** *P* < 0.0001.

A flow diagram was generated to systematically describe our study (**Figure 1A**). We collected 108 driver genes and 69 suppressor genes from FerrDb (http://www.zhounan.org/ferrdb), among which four genes were intersected, then 173 ferroptosis regulators were selected. Of the merged expression matrix containing 9 normal and 103 OS cell lines from GSE42352 and GSE36001, 143 of 173 ferroptosis regulators were detected. Subsequently, the expression of the 143 detected regulators were evaluated and visualized in heatmap, while 21 significant DEGs were identified (*P* < 0.05, |logFC| > 0.5) (**Figure 1B**). CBS, SCD, CDKN2A, SNX4, FANCD2, and HMGB1 were upregulated in OS, and 15 regulators, including PML, ACO1, MYB, NCOA4, ATG3, CDO1, SQSTM1, TNFAIP3, CDKN1A, CAV1, NQO1, TF, EPAS1, ZFP36, and AKR1C3 were downregulated. The Chi-square test indicated that the ratio of significant ferroptosis-related DEGs was statistically higher than that of other genes (**Figure 1C**). Therefore, these results indicated that the expression of ferroptosis regulators was dysregulated in OS.

### Ferroptosis regulators-based classification correlated with steosarcoma prognosis and immune microenvironment

**Figure 2:**
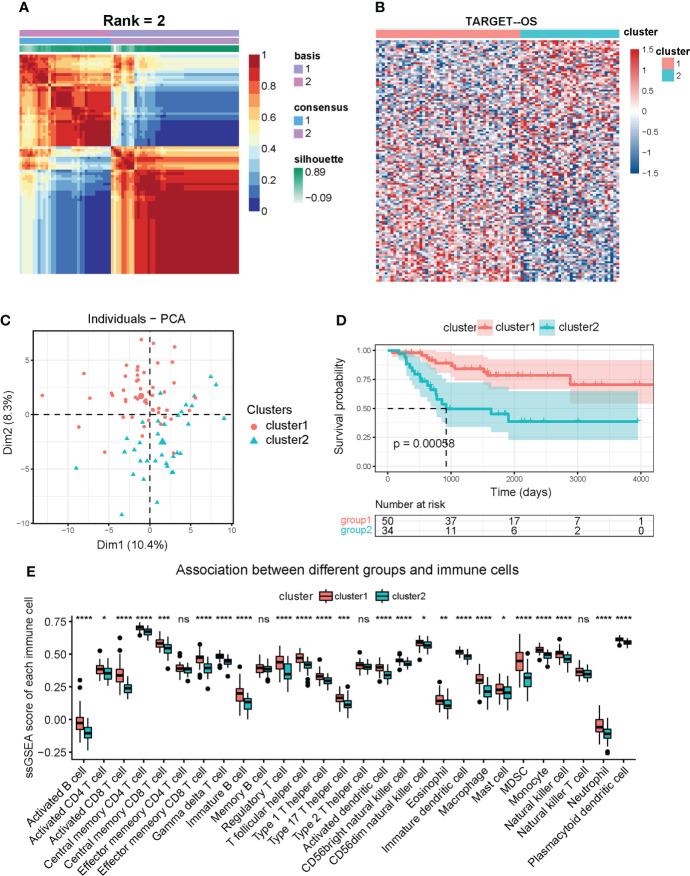
Identification of OS subclusters by unsupervised NMF clustering with ferroptosis regulators in TARGET OS cohort. **(A)** NMF clustering heatmap based on 132 ferroptosis genes (MAD > 0.5). NMF clustering divided 84 OS patients into two subclusters (we observed clearest boundary and most appropriate consistency when the coexistence correlation coefficient k = 2). **(B)** Heatmap of expression of the 132 ferroptosis regulators in two subclusters. Red represents high expression level and blue represents low expression. The darker the color, the greater the significance. **(C)** PCA scatter diagram supporting that NMF clustering algorithm divided OS patients into two subclusters. **(D)** Kaplan-Meier analysis for overall survival of OS patients in two subclusters. **(E)** Box diagram of ssGSEA analysis revealing expression of 28 immune cells in two subclusters. Kruskal test * *P* < 0.05; ** *P* < 0.01; *** *P* < 0.001; **** *P* < 0.0001; ns, no significance.

We downloaded TARGET-OS gene expression profiles from UCSC Xena (https://xenabrowser.net/) and screened out 84 patients with analyzable prognostic information as a training cohort. Based on the previously mentioned 173 ferroptosis-regulated genes, a total of 132 genes selected with MAD value > 0.5 (43–45) were applied for NMF clustering analysis. Then, unsupervised NMF clustering was performed to assess potential gene expression features by dividing the original matrix into subclusters. A comprehensive correlation coefficient determined the most appropriate k value. Compared with heatmaps at k values of 3, 4, and 5 (**Supplementary Figure 1A**), k = 2 generated a heatmap that displayed the clearest boundary and best consistency in every subcluster (**Figure 2A** and **Supplementary Figure 1B**). Thus 84 patients were clustered into two subclusters, 50 patients in cluster one and 34 patients in cluster two. The heatmap displays 132 selected ferroptosis regulators’ expression levels in clusters one and two (**Figure 2B**). PCA analysis was performed to verify the consistency of subcluster distribution (**Figure 2C**), which is highly consistent. Based on the clinical information of these patients in the TARGET cohort, survival analysis (**Figure 2D**) was constructed and revealed that cluster two OS patients exhibited poor survival outcomes compared with cluster one patients (p < 0.001). To investigate the 28 specific infiltrating immune cell types in tumor progression, ssGSEA was conducted (**Figure 2E**) and showed that cluster one was more positively correlated with immune cell infiltration than cluster two. The specific immune cells in this ssGSEA analysis included activated B cells, activated CD8 T cells, regulatory T cells, macrophages, NK cells, and others. Furthermore, based on the KEGG and GO databases, gene set variation analysis (GSVA) was performed to investigate the activation level of immune-related biological pathways in two subclusters (**Supplementary Figures 2A, B**). Our results demonstrated that cluster one is more relevant to various immune-related processes and pathways, such as NK cell-mediated cytotoxicity, primary immunodeficiency, T cell receptor signaling pathway, and regulation of macrophage fusion. Additionally, GO enrichment analysis was performed to comprehensively evaluate the biological characteristics in two ferroptosis-related subclusters and indicated that cluster one was closely correlated with immune-related activities (**Supplementary Figure 2C**), and cluster two was relevant to ion transmembrane channel activity and intercellular adhesion (**Supplementary Figure 2D**). In summary, these results suggest a significant difference in prognostic outcomes and biological characteristics within ferroptosis-related subclusters, and the difference in prognosis is highly correlated with the immune microenvironment.

### Construction of prognostic signature based on ferroptosis regulators in osteosarcoma cohort

**Figure 3:**
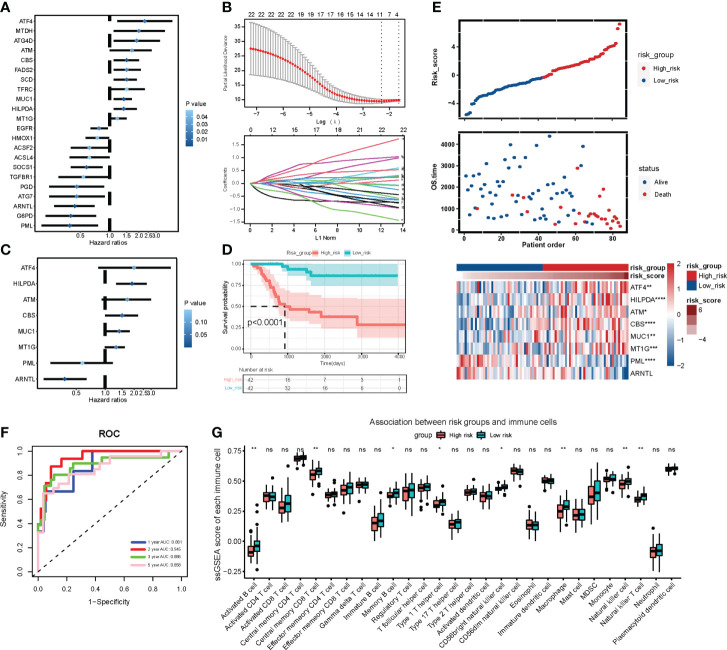
Construction and analysis of prognostic signature based on ferroptosis gene expression in TARGET OS cohort. **(A)** Forest plot of 22 independent prognostic genes identified by univariate Cox regression analysis (*P* < 0.05). Blue represents statistical significance. The deeper the blue, the greater the significance. **(B)** LASSO algorithm confirming minimum lambda value. **(C)** Forest plot of 8 regulators in optimal prognostic model selected by stepwise multivariate Cox regression analysis. **(D)** Kaplan-Meier analysis exhibiting the overall survival of OS patients in high-risk group and low-risk group graded by the optimal prognostic model. Red represents high risk group and blue represents low risk group. **(E)** Distribution plots of risk scores and heatmap of signature genes expression in TARGET OS patients. **(F)** Time dependent receiver operating characteristic (ROC) curve of the ferroptosis signature model in predicting prognosis of OS patients. **(G)** Box plot of ssGSEA analysis revealing 28 immune cells expression in two risk subgroups. Kruskal test * *P* < 0.05; ** *P* < 0.01; *** *P* < 0.001; **** *P* < 0.0001; ns, no significance.

Cox regression analysis and the LASSO regression algorithm were conducted to determine the prognostic value of ferroptosis regulators in OS. Among the OS patients in the TARGET cohort, initially, 22 independent prognosis-related genes were confirmed by univariate Cox regression analysis (*P* < 0.05) (**Figure 3A**). Then, the LASSO algorithm filtered 11 ferroptosis regulators that met the minimum lambda value from the 22 genes (**Figure 3B**). Based on the LASSO results, stepwise multivariate Cox regression analysis was performed to construct a prognostic signature model (**Figure 3C**), which selected an optimal model containing eight genes: ATF4, HILPDA, ATM, CBS, MUC1, MT1G, PML, and ARNTL. Subsequently, every patient obtained a risk score calculated based on the eight regulators’ regression coefficients and expression levels. Patients were classified into high-risk and low-risk groups using the median risk score (46, 47). The Kaplan-Meier analysis revealed that patients in the high-risk group exhibit poor overall survival compared with low-risk group patients (*P* < 0.0001) (**Figure 3D**). The expression of the eight risk genes is shown in the heatmap (**Figure 3E**). The Scatter diagram displayed that the high-risk group correlated more with death incidents (**Figure 3E**). Time-dependent ROC (**Figure 3F**) indicated that the area under the curve (AUC) of 1-year, 2-year, 3-year, and 5-year survival was 0.881, 0.945, 0.886, and 0.858, respectively. Notably, it was most accurate for the risk score to predict 2-year survival. Additionally, Kaplan-Meier analyses based on these 8 genes respectively verified their potential to serve as independent prognosis factors (**Supplementary Figures 3A–H**). These results suggest the potential value of the constructed risk signature in predicting the prognosis of OS patients.

**Figure 4:**
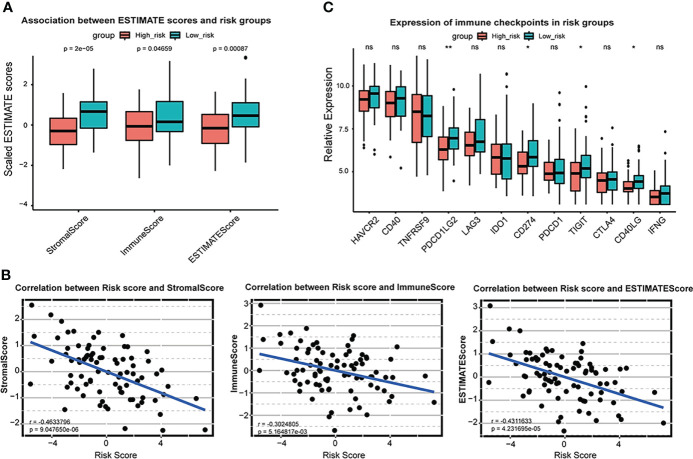
Association between ESTIMATE scores and risk scores in TARGET OS cohort. **(A)** Box plot of Stromal score, immune score and ESTIMATE score and in high-risk group and low-risk group. Red represents high risk group and blue represents low risk group. **(B)** Scatter plot shows correlation between risk score and stromal score, immune score and ESTIMATE score. **(C)** Box plot of relative expression of immune checkpoints in risk groups. * *P* < 0.05; ** *P* < 0.01; ns, no significance.

Moreover, ssGSEA analysis revealed that the high-risk group was likely to have less expression of immune cells, including activated B cells, macrophages, and NK cells (**Figure 3G**). In ESTIMATE analysis, Stroma, Immune, and ESTIMATE scores were prominently lower (T-test *P* < 0.05) in the high-risk group than those in the low-risk group (**Figure 4A**). Correlation analysis revealed that risk score was negatively correlated with Stromal, Immune, and ESTIMATE scores (**Figure 4B**). Relative expression of immune checkpoints in two risk groups was also visualized (**Figure 4C**), in which checkpoints PDCD1LG2, CD274, TIGIT, and CD40LG were observed at relatively low levels in a high-risk group. These results suggest that the risk score based on the ferroptosis prognostic signature was associated with immunosuppression and tumor progression.

In the validation set from GSE21257, a prognostic signature was executed to calculate risk scores. Then, 53 OS patients were divided into high-risk and low-risk groups using the median risk score. The heatmap demonstrated expression of the eight risk genes (**Supplementary Figure 4A**), and the scatter diagram indicated increased death incidents in the high-risk group (**Supplementary Figure 4A**). The Kaplan-Meier analysis revealed that high-risk patients possessed poor overall survival compared with low-risk patients (*P* < 0.05) (**Supplementary Figure 4B**). Additionally, time-dependent ROC (**Supplementary Figure 4C**) indicated that the area under the curve (AUC) of 1-year, 2-year, 3-year, 5-year, and 8-year survival was 0.658, 0.694, 0.754, 0.718, and 0.689, respectively.

### Single cell sequencing investigated the relevance between risk stratification and immunity

**Figure 5:**
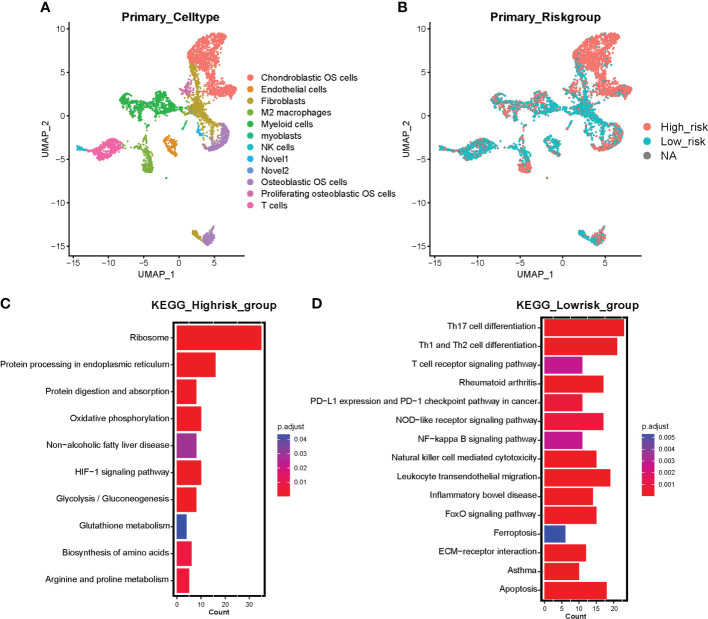
Single-cell sequencing investigating the correlation between risk signature and tumor microenvironment in primary OS samples. **(A)** UMAP visualization exhibits 12 annotated cell clusters based on primary OS single cell sequencing. **(B)** Risk cell clustering by ferroptosis signature clusters all cells into high-risk cells and low-risk cells. Red represents high-risk cells and blue represents low-risk cells. NA represents partial signature genes were not expressed in the single-cell sparse matrix. **(C)** KEGG enrichment analysis based on marker genes of high-risk cells. **(D)** KEGG enrichment analysis based on marker genes of low-risk cells. Color represents adjusted p value (Benjamini-Hochberg), the darker the red, the higher the significance; the darker the blue, the lower the significance.

To further investigate the correlation between ferroptosis risk signature and immune infiltration in OS, we collected scRNA-seq expression profile from GSE152048 on the GEO database, containing primary, lung metastatic, and recurrent OS lesions. Firstly, 16 cell subclusters were identified by “UMAP” dimension reduction in primary OS lesions expression profile (**Supplementary Figures 5A, B**). Expression proportions of the eight signature genes among all detected cells in primary OS samples were also visualized (**Supplementary Figures 5C**, **4D**), in which we observed a relatively high proportion in the expression of ATF4, HILPDA, and ATM. All subclusters were annotated with feature genes and visualized into 12 cell clusters, including chondroblastic OS cells, endothelial cells, fibroblasts, M2 macrophages, myeloid cells, NK cells, osteoblastic OS cells, proliferating osteoblastic OS cells, T cells, and novel 1 and novel 2 (**Figure 5A**). Then, the prognostic signature was applied to calculate the risk scores of all cells and divided into high-risk and low-risk cells by median risk score. We found that chondroblastic OS cells, osteoblastic OS cells, proliferating osteoblastic OS cells, a subset of M2 macrophages, and myeloid cells were identified as high-risk cells, and immune cells, including T cells and NK cells, were identified as low-risk cells (**Figure 5B**). Subsequently, marker genes in high-risk cells and low-risk cells were distinguished by the “FindMarkers” function of the “Seurat” R package. KEGG enrichment analysis based on these markers indicated that high-risk cells were correlated with several cancer-related pathways, including oxidative phosphorylation, HIF-1 signaling pathway, and glycolysis/gluconeogenesis (**Figure 5C**). In contrast, low-risk cells were associated with immune-related pathways, including the T cell receptor signaling pathway, PD-L1 expression and PD-1 checkpoint pathway in cancer, NF-κB signaling pathway, NK cell-mediated cytotoxicity, and others (**Figure 5D**). Moreover, the low-risk group was correlated with ferroptosis and apoptosis (**Figure 5D**). Notably, these results support a risk score based on constructed prognostic signatures positively associated with tumor progression and negatively associated with immune infiltration and programmed cell death like ferroptosis and apoptosis in primary OS lesions.

Additionally, 13 cell subclusters were identified in metastatic OS lesions expression profile (**Supplementary Figures 6A, B**). The proportion diagram exhibited a markedly elevated expression level of MT1G compared with primary OS lesions (**Supplementary Figures 6C, D**). Moreover, all subclusters were annotated and visualized into 10 cell clusters, including chondroblastic OS cells, endothelial cells, fibroblasts, M2 macrophages, myoblast, NKT/T cells, osteoblastic OS cells, osteoclasts, proliferating osteoblastic OS cells and B cells (**Supplementary Figure 6E**). Then the prognostic signature was performed to calculate risk scores and divided all cells into high-risk and low-risk groups. Chondroblastic OS cells, osteoblastic OS cells, and proliferating osteoblastic OS cells were defined as high-risk cells, and B cells, NK T cells, and T cells were low-risk cells (**Supplementary Figure 6F**). Six-cell clusters were annotated and visualized in recurrence OS lesions, including chondroblastic OS cells, fibroblasts, myeloid cells, NKT/T cells, and novel cells. Prognostic signature classified all recurrence cells into high-risk and low-risk groups, indicating that chondroblastic OS cells, osteoblastic OS cells, and a part of fibroblasts were high-risk cells, and NKT/T cells were low-risk cells (**Supplementary Figures 7A–F**). The proportion diagram exhibited the up-regulation of HILPDA, MUC1, and MT1G in recurrence OS lesions. Therefore, these findings suggest a vital role of five cancer-promoting genes: ATF4, HILPDA, ATM, MUC1, and MT1G in affecting the OS progression, metastasis, and recurrence.

### Knocking down of HILPDA or MUC1 significantly inhibited the proliferation of OS cells

**Figure 6:**
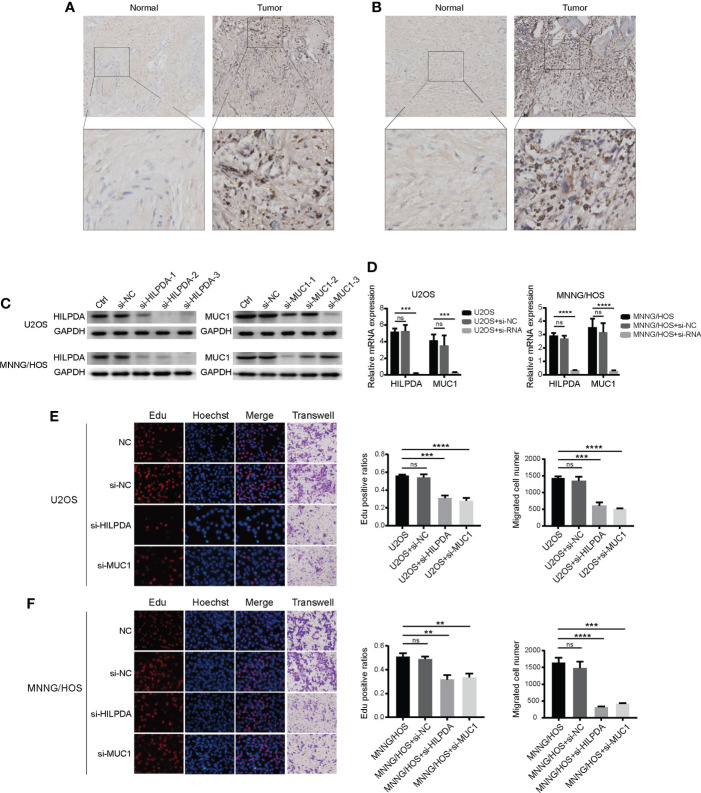
Expression of HILPDA or MUC1 influenced the proliferation of OS cells. Representative immunohistochemical images of expressions of HILPDA **(A)** and MUC1 **(B)** in OS and para-carcinoma tissues. **(C)** Relative protein levels of HILPDA and MUC1 after si-RNA transfection (n=3) in U2OS and MNNG/HOS. **(D)** Relative mRNA expression levels of HILPDA and MUC1 using the optimal si-RNA **(E, F)** Representative images of EdU (red), Hoechst staining (blue) and transwell (purple) in U2OS and MNNG/HOS cells after si-RNA transfection.The ratios of EdU-positive (red) cells and migration cell numbers were calculated (n=3) after si-RNA transfection. Student t test ** *P* < 0.01; *** *P* < 0.001; **** *P* < 0.0001; ns, no significance.

We further analyzed the five cancer-promoting prognostic genes and found that ATF4 (48), ATM (49), and MT1G (50) have been reported in OS, while the functions of HILPDA and MUC1 remained unclear. We then chose HILPDA and MUC1 as the following research subjects to illustrate their functions in OS. The expression levels of HILPDA (**Figure 6A**) and MUC1 (**Figure 6B**) were upregulated in OS tissues compared with paracancerous normal tissues. Then we used small interfering RNA to silence the expression of HILPDA and MUC1 in two OS cell lines. In U2OS cells, si-HILPDA sequence-2 and si-MUC1 sequence-3 had the best interference effect, while in MNNG/HOS cells, si-HILPDA sequence-3 and si-MUC1 sequence-1 were the optimal (**Figures 6C, D**). Correspondingly, compared to normal control groups, the percentages of Edu-positive OS cells and migrated cell numbers were significantly reduced in si-HILPDA and si-MUC1 groups (**Figures 6E, F**). The proliferation and migration of OS cells were inhibited considerably after interfering with HILPDA or MUC1 expression.

### HILPDA and MUC1 influenced ferroptosis resistance of OS cells

**Figure 7:**
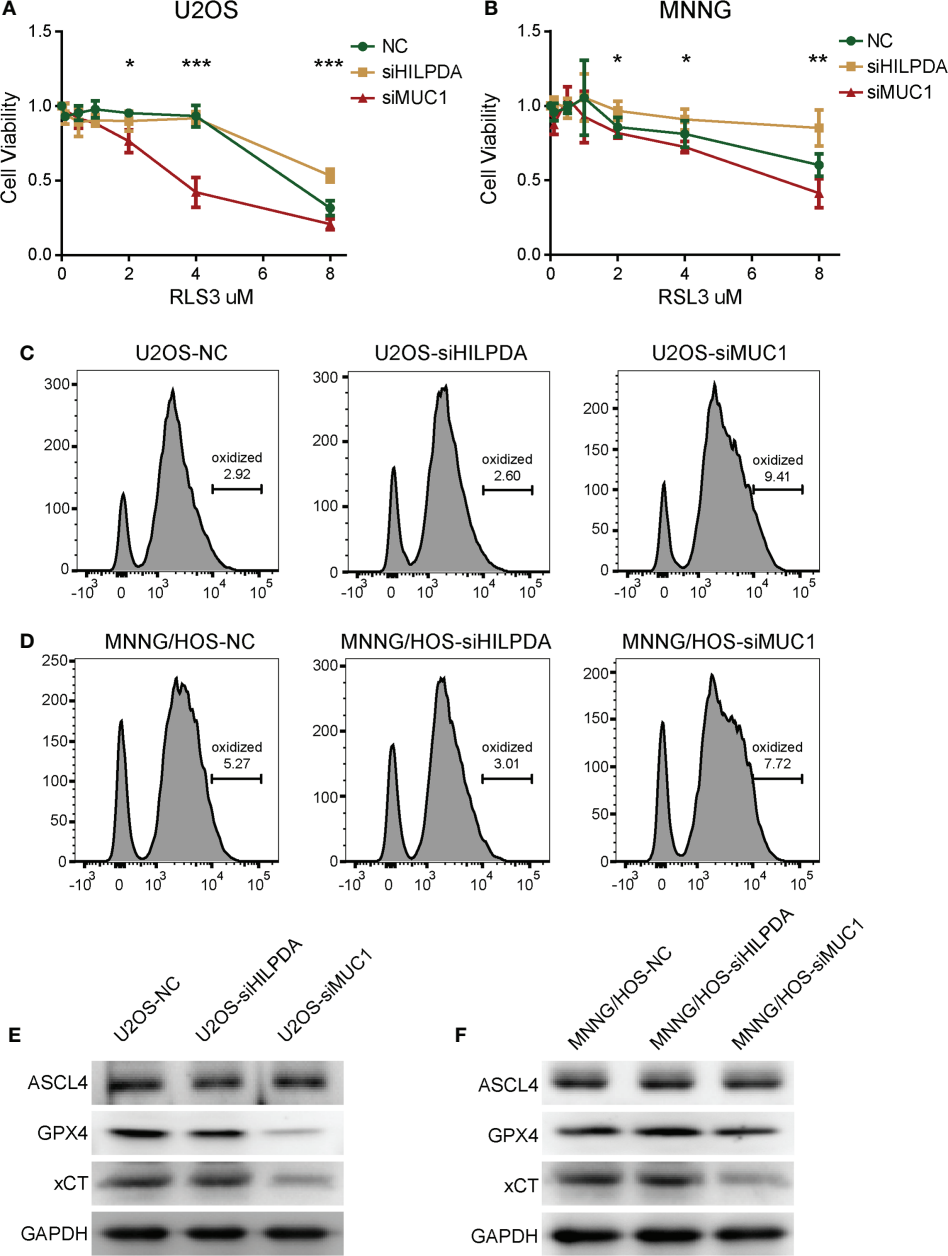
The effects HILPDA or MUC1 on ferroptosis resistance in OS cells. CCK-8 method detected the cell viability of U2OS **(A)** and MNNG/HOS **(B)** after treatment by different concerntrations of RSL3 for 24h. One-way Anova test *P< 0.05; **P< 0.01; ***P< 0.001. Lipid ROS levels of RSL3 induced U2OS **(C)** and MNNG/HOS **(D)** after C11 BODIPY incubation based on flow cytometry. Levels of ferroptosis-related proteins including ASCL4, GPX4 and xCT in RSL3 induced U2OS **(E)** and MNNG/HOS **(F)**.

**Figure 8:**
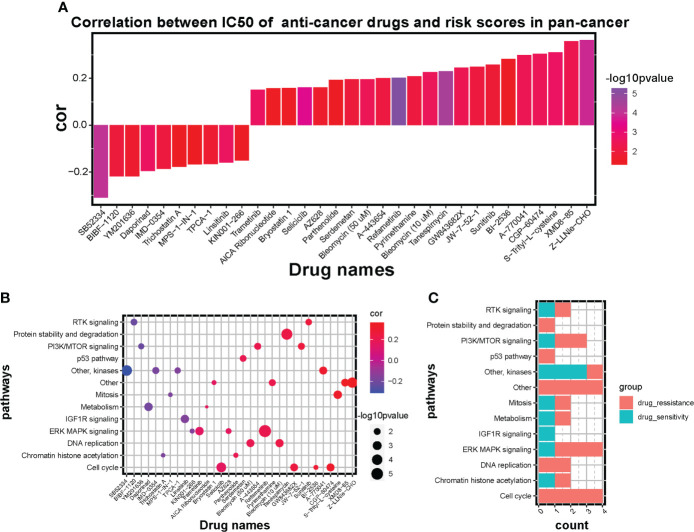
Influence of the risk signature on drug sensitivity and resistance in GSDC pan-cancer cell lines. **(A)** Bar diagram of correlation between IC50 of anti-cancer drugs and risk scores in pan-cancer. Altitude represents the correlation, higher the altitude, higher the correlation. Color represents statistical significance (p value), the more purple the color, the greater the significance. **(B)** Scatter diagram of correlation between targeted signaling pathways and IC50 of significant anti-cancer drugs. Size of plots represents statistical significance (p value), the larger the size, the greater the significance. Color of plots represents the correlation between targeted pathways and anti-cancer drugs. Red represents positive correlation and blue represents negative correlation. Purple represents little correlation. **(C)** Bar diagram shows the counts of sensitive drugs and resistant drugs regarding the targeted pathways.

Both HILPDA and MUC1 were reported ferroptosis-related regulators, but mechanisms of how they affect ferroptosis remain to be further investigated. In our subsequent experiments, we used gradient concentration of ferroptosis inducer RSL3 to treat U2OS and MNNG/HOS, and 24h later, the CCK-8 method was used to detect the cell viability. Compared with the control group, the si-MUC1 group exhibited poor cell viability. The si-HILPDA group had a higher survival rate, with the greatest difference when RSL3 concentration was 4μM in U2OS (**Figure 7A**) and 8μM in MNNG/HOS (**Figure 7B**). Thus, U2OS with 4μM RSL3 and MNNG/HOS with 8μM treatment were used for subsequent experiments. Based on Flow Cytometry, the lipid ROS level was increased in the si-MUC1 group and decreased in the si-HILPDA group (**Figures 7C, D**), indicating RSL3-induced activity was correlated with lipid peroxidation, the marker of ferroptosis. We further assessed the levels of several ferroptosis-related proteins (**Figures 7E, F**). Among the control, si-HILPDA, and si-MUC1 groups, ASCL4 exhibited no significant difference, and xCT was decreased in the si-MUC1 groups. Intriguingly, GPX4 seemed to decrease in the si-MUC1 group of U2OS cells while slightly upregulated in the si-HILPDA group of MNNG/HOS cells. This finding might explain the earlier appearance of RSL3-induced ferroptosis in si-MUC1 U2OS cells and the enhanced ferroptosis resistance in si-HILPDA MNNG/HOS cells.

### Predictive value on therapeutic strategies of the prognostic signature

To explore the potential value of risk signature in predicting therapeutic strategies, based on the Genomics of Drug Sensitivity in Cancer (GSDC) database, spearman analysis was performed to investigate the correlation between half-maximal inhibitory concentration (IC50) of drugs and risk scores in cancer cell lines. A total of 32 drugs were identified to be significantly associated with the prognostic signature score (|cor| > 0.15, *P* < 0.05) (**Figure 8A**). Among them, drug sensitivity of 10 drugs were determined relevant to the score, including RTK signaling inhibitor BIBF 1120 (cor = -0.22, *P* = 0.012), PI3K/mTOR signaling inhibitor YM201636 (cor = -0.22, *P* = 0.019) and IGF1R signaling inhibitor Linsitinib (cor = -0.16, *P* = 0.002). However, drug resistance of 22 drugs were correlated with risk score, including cell cycle inhibitor CGP-60474 (cor = 0.31, *P* = 0.003), RTK signaling inhibitor Sunitinib (cor = 0.26, *P* = 0.013), and DNA replication inhibitor Bleomycin (cor = 0.23, *P* = 0.003). Additionally, targeted signaling pathways of these drugs were exhibited (**Figures 8B, C**) and indicated that drugs whose sensitivity was positively related to risk scores mostly target RTK signaling, kinases, IGF1R signaling, and ERK MAPK signaling. However, drugs whose resistance was positively related to risk scores targeted PI3K/mTOR signaling, ERK MAPK signaling, DNA replication, and cell cycle signaling. Therefore, established risk signatures might serve as potential guidance for establishing therapeutic strategies.

## Discussion

Therapeutic schedules and outcomes of OS patients have remained significantly unimproved since the 1970s (3). Despite decades of research, molecular exploration still needs to diagnose the disease early, predict the progression and improve the prognosis for OS, especially for lung metastasis and chemotherapy resistance patients (51). Yanlong et al. found that focally amplified long noncoding RNA (lncRNA) expression on chromosome 1 (FAL1) was positively related to the distance metastasis, tumor stage, and negatively prognosticate outcomes in OS patients (52). Wei et al. also showed that cyclin E1 was a promising prognostic and chemotherapeutic target for OS (53). These studies promoted the potential and significance of exploring molecular biomarkers for the onset and development of OS. In addition to these molecular biomarkers, ferroptosis has been considered a promising antitumor target mechanism in the occurrence and progression of numerous cancers (13, 54, 55). Evidence suggests multiple risk signatures based on ferroptosis genes could effectively predict the diagnosis, prognosis, immune microenvironment, and therapeutic strategies for cancers (56–58). However, few studies reported the correlation between ferroptosis mechanisms and OS progression.

Our study initially showed the dysregulation of ferroptosis regulators with normal and patient-derived OS cell lines, which was statistically significant by the Chi-square test (**Figure 1**). Whereas biological deviations existed between specific DEGs in cell lines and RNA sequencing results from OS patients, we set MAD > 0.5 as gene screening criteria for following NMF clustering analysis instead of using DEGs from OS cell lines. In doing so, two distinct subclusters were identified with different biological characteristics (**Figure 2**). OS patients in cluster1 exhibited a more favorable prognosis than those in cluster2, indicating that identified subclusters had significant clinical implications. Meanwhile, ssGSEA and GSVA for immune pathways and GO enrichment analyses suggested a higher degree of immune cell expression and immune response activity in cluster1 (**Supplementary Figure 2**). Existing studies indicate an association between immune response and tumor progression. Chi et al. (59) revealed that NK T cells promoted antitumor immunity in liver tumors. Mary et al. (60) found that the dysregulation of CD8 T cells would allow for tumor progression. Moreover, the Toll-like receptor signaling pathway benefits immune-related anticancer chemotherapy and radiotherapy (61). Our results are consistent with these dominant perceptions that the immune microenvironment’s abundance correlates with better clinical outcomes. Given the above discovery, we speculated that cluster1, having a better prognosis, was more immune-activated than cluster2, and ferroptosis was involved in shaping the immune microenvironment in OS.

Considering the heterogeneity and complexity of individuals, we constructed a risk scoring system, “ferroptosis-based risk signature,” to quantify the biological characteristics of OS patients (**Figure 3**). High-risk scores with worse clinical outcomes exhibited strong relevance to immunosuppression and lower stromal scores (**Figure 4**). The significance of immune and stromal scores in the ESTIMATE algorithm for tumor classification and clinical outcomes was already testified (62, 63). Hence we speculated that our constructed ferroptosis score was more significant in predicting immunosuppression than in predicting the stromal activation for OS malignancy. Moreover, immune checkpoints like PDCD1LG2, CD274, TIGIT, and CD40LG were upregulated in low-risk groups, reflecting the potential of immunotherapy in managing OS (**Figure 4C**). These results suggest that ferroptosis-based risk signature is reliable for comprehensively predicting the clinical prognosis, immune response activity, and therapeutic strategy for OS.

Among the 8 independent prognosis factors (**Supplementary Figure 3**) in the signature, ATF4, HILPDA, ATM, CBS, MUC1, and MT1G were significantly upregulated in the high-risk group, whereas PML was down-regulated (**Figure 3E**), implying that PML might serve as an antineoplastic factor in OS progression. Chen et al. (64) found that expression of activating transcription factor 4 (ATF4) promoted the malignancy of gliomas and fostered tumor angiogenesis and proliferation, while ATF4 knockdown made cells susceptible to ferroptosis. Hypoxia-inducible lipid droplet-associated (HILPDA) (65, 66) was overexpressed in multiple tumor types, HILPDA was positively correlated with tumor-associated macrophages (TAM) infiltration, and immunosuppressive genes, such as PD-L1, PD-1, TGFB1, and TGFBR1. Notably, Ataxia-Telangiectasia mutated protein (ATM) was reported as a positive regulator for ferroptosis (67). Radiotherapy-activated ATM and IFNγ from immunotherapy-activated CD8+ T cells would synergistically enhance ferroptosis and tumor lipid oxidation, indicating the correlation between ferroptosis agonists and chemoradiotherapy *via* immunotherapy for the first time (68). Li Wang et al. (69) found that inhibition of Cystathionine β-synthase (CBS) triggered ferroptosis in hepatocellular carcinoma and reduced tumor growth. Takahiro et al. (70) showed that the transmembrane mucin MUC1 contributed to immunologic escape in triple-negative breast cancer (TNBC) and that targeting MUC1-C correlated with PD-L1 suppression to activate the immune response and tumor cell killing. Emerging evidence suggests the crucial role of metallothioneins (MTs), including MT1G, in tumor formation, progression, and drug resistance (71). As a tumor suppressor, promyelocytic leukemia (PML) protein was mechanistically capable of inhibiting tumor proliferation, migration, and invasion while promoting cell senescence and apoptosis (72–74). A recent study also reported that ubiquitination of PML promotes lung cancer progression *via* fostering immunosuppression in the tumor microenvironment (75).

Single-cell sequencing analysis further investigated the role of ferroptosis signature in the tumor microenvironment and malignant cell proliferation of OS (**Figure 5**). Neoplastic cells and M2 macrophages were identified in the high-risk group, while immune cells were mostly identified in the low-risk group. Growing evidence has clarified the crucial role of TAMs in the progression and metastasis of tumors (76, 77). Additionally, Zhou et al. found the preventive effect of inhibiting M2 polarization of TAMs in OS metastasis (78). Moreover, previous work indicated that the infiltration degree of intratumoral T cells was positively effective in predicting the prognosis of colorectal cancer, ovarian cancer, and melanoma patients (79–81). Existing research also reported the cytotoxic effect of NK cells against tumor progression in multiple cancers (82, 83). Our results implied that the prognostic signature could predict tumor invasion and progression from the M2 polarization of TAMs. The risk score was negatively correlated with anticancer immune cell infiltration in primary OS. However, in the high-risk group, several cancer-promoting pathways were enriched (**Figure 5C**). Oxidative phosphorylation is upregulated in multiple cancers, including leukemias, melanoma, pancreatic ductal adenocarcinoma lymphomas, and endometrial carcinoma (84). Similarly, high-rate glycolysis can promote tumor proliferation in an aerobic environment (85). Importantly, HIF-1 functions as a crucial signal by coordinating tumorigenesis-related transcription factors and signaling molecules (86); Ni et al. suggests that inhibition of HIF-1α would unleash the activity of tumor-infiltrating NK cells (87). In the low-risk group, immune-related pathways were enriched as expected, including the T cell receptor signaling pathway, PD-L1 expression and PD-1 checkpoint pathway in cancer, NF-κB signaling pathway, and NK cell-mediated cytotoxicity. However, ferroptosis and apoptosis were also correlated with low-risk cells (**Figure 5D**), suggesting that ferroptosis risk score was negatively relevant to ferroptosis occurrence and ferroptosis occurrence in OS cells associated with immune system activation.

Based on the above findings, we chose two prognostic genes to illustrate our results through functional experiments in OS cells. HILPDA and MUC1 expression were verified to be increased in OS tissues (**Figures 6A, B**), and we confirmed the knockdown of HILPDA or MUC1 could inhibit the proliferation and migration of OS cells (**Figure 6**). Notably, Hasegawa et al. reported that MUC1-C forms a complex with xCT, which interacts with xCT and thereby controls GSH levels (88) and that xCT activity drives the expression GPX4 (89). Our results showed that interference targeting MUC1 led to the decrease of xCT, and GPX4 also exhibited downregulation. Therefore, the decline in these two anti-ferroptosis proteins (89) might be the potential mechanism of weakened ferroptosis resistance in MUC1-knockdown cells (**Figure 7**). However, HILPDA-knockdown cells seemed to have enhanced ferroptosis resistance (**Figure 7**). Thus, the restraint in OS invasiveness regarding HILPDA knockdown is probably unrelated to the ferroptosis mechanism.

Adverse chemotherapy combined with surgical removal of OS lesions is the primary management strategy for OS patients (3), while chemoresistance has become a pivotal obstacle in improving the therapeutic effect (90). The interaction between ferroptosis and chemoresistance has recently been a topic of investigation, which Zhang et al. (91) reports that cisplatin and paclitaxel facilitated the secretion of miR-522 from cancer-associated fibroblasts, leading to ALOX15 suppression, ferroptosis inhibition, and ultimately chemoresistance. Our analysis for IC50 of anticancer drugs (**Figure 8**) showed the potential therapeutic efficiency of ferroptosis regulators. The ferroptosis risk score was correlated with sensitivity to drugs targeting RTK, IGF1R signaling, and kinases and with resistance to drugs targeting PI3K/mTOR, ERK/MAPK signaling, DNA replication, and cell cycle signaling. These results imply that patients with higher ferroptosis scores may benefit more from chemotherapy drugs targeting RTK, IGF1R signaling, and kinases. Ferroptosis regulators might be an adequate predictor for evaluating chemoradiotherapy’s prognosis or targeted therapies. Therefore, our findings provided new probabilities for improving the management strategies for OS.

There are still some limitations in our study. Firstly, the data capacity for OS in public databases is significantly less than that for other tumor types, obstructing the exploration of OS bioinformatics research. To enlarge the sample capacity of the control group, we extracted control cell lines with inconsistent standards to accomplish the variation analysis, which could result in unpredictable biological deviations. More practicable sequencing data is yet to be discovered. Likewise, the interaction between stromal cell and ferroptosis signature remains unclear, as well as the major function of stromal cells in tumor progression and infiltration. Secondly, checkpoint PD-1/PD-L1 (CD274) has been reported as a pivotal mediator of immunosuppression in the tumor immune microenvironment (92, 93). Zheng et al. (94) demonstrated that PD-L1 was negatively associated with prognosis, while PD-L2 (PDCD1LG2) positively correlated with overall survival in OS. Given our contradictory result that the expression level of checkpoint CD274 was higher in the low-risk group, further inquiry about the molecular mechanisms of CD274 affecting ferroptosis signature and OS prognosis is needed, and PD-L1-related immune therapy on OS remains to be developed. Thirdly, ferroptosis-related gene signature for OS is not a novel subject. Lei et al. (95), Zhao et al. (96), Jiang et al. (97) all reported prognostic ferroptosis signatures, which might make our finding less novel. However, our study appears to be the first to reveal the correlation between immune landscape and ferroptosis signature from the perspective of a single-cell sequence. Notably, we are the first to propose the potential ferroptosis mechanism of specific genes, HILPDA and MUC1 regarding ferroptosis signature. From mechanistic investigations, we confirmed the cancer-promoting function of HILPDA and MUC1. However, the potential mechanisms or detailed pathways between HILPDA, MUC1, and ferroptosis require further exploration. Furthermore, the specific roles of the other six genes and their crosslinking remains to be explored. Generally, existing data and results could only support the predicting value of ferroptosis signature on OS progression, immune activity, and patient prognosis. The activation mechanism of ferroptosis signature to intervene in the immune system is lacking. Therefore, more experiments are needed to explore the mechanism of ferroptosis signature in OS immunology.

## Conclusions

In summary, our study comprehensively evaluated the expression pattern and prognostic value of ferroptosis regulators in OS. Our study’s constructed prognostic model based on ferroptosis regulators is promising in predicting tumor progression, immune infiltration, and survival outcome of OS patients. Moreover, the risk stratification had a guidance value on chemoradiotherapy and might be correlated with the efficacy of immunotherapy. We also confirmed the cancer-promoting function of HILPDA and MUC1 and the ferroptosis-resistant related mechanism of MUC1 in OS, which suggested that MUC1 has the potential to become a ferroptosis-related therapeutic target. However, further exploration is necessary to reveal the potential mechanism among these genes in OS progression and therapeutic efficacy.

## Data availability statement

The original contributions presented in the study are included in the article/**Supplementary Material**. Further inquiries can be directed to the corresponding author.

## Ethics statement

The studies involving human participants were reviewed and approved by the institutional review board (IRB) of the Third Xiangya Hospital, Central South University (No: 2020-S221). Written informed consent for participation was not required for this study in accordance with the national legislation and the institutional requirements.

## Author contributions

Conception and design: XW, XC and SW. Foundation support: XC and SW. Acquisition and analysis of data: XW. Cell experiment and analysis: XW, LZ and GX. Interpretation of data: XW and SX. Drafting the manuscript and revising for submission quality: XW and XC. Reviewing and approving the final vision: XW, JH, WZ and XC. Study supervision: SW and XC. All authors contributed to the article and approved the submitted version.

## Funding

This work was financially supported by the National Natural Science Foundation of China (82072501), Science and Technology Innovation Leading Plan of High-Tech Industry in Hunan Province (2020SK2011), Youth Fund Project of Natural Science Foundation of Hunan Province (2020JJ5848), and Medical Research Development Fund Project (WS865C).

## Acknowledgments

The authors thank AiMi Academic Services (www.aimieditor.com) for the English language editing and review services.

## Conflict of interest

The authors declare that the research was conducted in the absence of any commercial or financial relationships that could be construed as a potential conflict of interest.

## Publisher’s note

All claims expressed in this article are solely those of the authors and do not necessarily represent those of their affiliated organizations, or those of the publisher, the editors and the reviewers. Any product that may be evaluated in this article, or claim that may be made by its manufacturer, is not guaranteed or endorsed by the publisher.

## Supplementary material

Supplementary Figure 1 Unsupervised consensus NMF clustering on TARGET cohort. **(A)** Heatmaps of NMF clustering for k = 3, 4, and 5. **(B)** The correlation among cophenetic, dispersion, evar, residuals, rss, silhouette and sparseness coefficients with reference to different cluster number.

Supplementary Figure 2 Enrichment characteristics in two ferroptosis subclusters. **(A, B)** GSVA analysis for NMF clustered ferroptosis subclusters based on KEGG database and GO database. Red represents high expression level and blue represents low expression. The darker the color, the greater the significance. **(C, D)** GO enrichment analysis including biological process, molecular function and cellular component based on high-expression genes in cluster one and cluster two. The length of bars represents gene counts of GO terms. Color represents adjusted p value (Benjamini-Hochberg), the redder the color, the greater the significance.

Supplementary Figure 3 Kaplan–Meier survival analysis for TARGET OS patients based on the expression of ATF4 **(A)**, HILPDA **(B)**, ATM **(C)**, CBS **(D)**, MUC1 **(E)**, MT1G **(F)**, PML **(G)**, ARNTL **(H)**.

Supplementary Figure 4 Validation of the ferroptosis prognostic signature in GEO OS cohort. **(A)** Distribution plots of risk scores and heatmap of signature gene expression in GEO OS patients. Red represents high expression level and blue represents low expression. The darker the color, the greater the significance. **(B)** Kaplan-Meier analysis exhibiting the overall survival of GEO OS patients in high-risk group and low-risk group. **(C)** Time dependent ROC curve analysis of the ferroptosis signature model in predicting prognosis of GEO OS patients. * *P* < 0.05; ** *P* < 0.01; *** *P* < 0.001; **** *P* < 0.0001.

Supplementary Figure 5 Single cell sequencing analysis of primary OS samples. **(A)** UMAP visualized 16 cell subclusters identified using “FindClusters” function. **(B)** Heatmap of top10 feature genes in 16 subclusters. Yellow represents high expression level of genes, purple represents low expression. **(C)** Expression of eight risk signature genes in all identified cells. Purple represents high expression of signature genes. The more purple the color, the higher the expression. **(D)** Expression proportion of eight signature genes among all detected cells in primary OS samples. Red represents the proportion of gene-positive cells and blue represents the proportion of gene-negative cells.

Supplementary Figure 6 Single cell sequencing analysis of metastatic OS samples. **(A)** UMAP visualized 13 cell subclusters identified in metastatic OS samples using “FindClusters” function. **(B)** Heatmap of top10 feature genes in the 13 subclusters. Yellow represents high expression level of genes, purple represents low expression. **(C)** Expression of eight risk signature genes in all identified cells. Purple represents high expression of signature genes. The more purple the color, the higher the expression. **(D)** Expression proportion of eight signature genes among all detected cells in metastatic OS samples. Red represents the proportion of gene-positive cells and blue represents the proportion of gene-negative cells. **(E)** UMAP visualization exhibits 10 annotated cell clusters based on metastatic OS single cell sequencing. **(F)** Risk cell clustering by ferroptosis signature clusters all cells into high-risk cells and low-risk cells. Red represents high-risk cells and blue represents low-risk cells. NA represents partial signature genes were not expressed in the single-cell sparse matrix.

Supplementary Figure 7 Single cell sequencing analysis of recurrent OS samples. **(A)** UMAP visualized 13 cell subclusters identified in recurrent OS samples using “FindClusters” function. **(B)** Heatmap of top10 feature genes in the 13 subclusters. Yellow represents high expression level of genes; purple represents low expression. **(C)** Expression of eight risk signature genes in all identified cells. Purple represents high expression of signature genes. The more purple the color, the higher the expression. **(D)** Expression proportion of eight signature genes among all detected cells in recurrent OS samples. Red represents the proportion of gene-positive cells and blue represents the proportion of gene-negative cells. **(E)** UMAP visualization exhibits 6 annotated cell clusters based on recurrent OS single cell sequencing. **(F)** Risk cell clustering by ferroptosis signature clusters all cells into high-risk cells and low-risk cells.

## Abbreviations

OS, osteosarcoma; TARGET, Therapeutically Applicable Research to Generate Effective Treatments; GEO, Gene Expression Omnibus; NMF, Non-negative matrix factorization; MAD, Median absolute deviation; PCA, Principal component analysis; GSVA, Gene set variation analysis; GO, Gene Ontology; KEGG, Kyoto Encyclopedia of Genes and Genomes; TME, tumor microenvironment; ssGSEA, single-sample gene-set enrichment analysis; ESTIMATE, Estimated Stromal and Immune cells in Malignant Tumor tissues using Expression; LASSO, least absolute shrinkage and selection operator; HR, Hazard ratio; UMAP, Uniform Manifold Approximation and Projection for Dimension Reduction; siRNA, Small interfering RNA; RT-qPCR, Real-time quantitative polymerase chain reaction; GSDC, Genomics of Drug Sensitivity in Cancer; IC50, half-maximal inhibitory concentration; ROC, Receiver operating characteristic; AUC, area under the curve; DEGs, differentially expressed genes; GAPDH, glyceraldehyde-3-phosphate dehydrogenase; ATF4, activating transcription factor 4; HILPDA, hypoxia inducible lipid droplet associated; ATM, ATM serine/threonine kinase; CBS, cystathionine beta-synthase; MUC1, mucin 1, cell surface associated; MT1G, metallothionein 1G; PML, PML nuclear body scaffold; ARNTL, aryl hydrocarbon receptor nuclear translocator like.
