# Supplementary figures and images for: A ferroptosis-related gene signature associated with immune landscape and therapeutic response in osteosarcoma
Source: Front Oncol. 2022 Nov 11;12:1024915. doi: 10.3389/fonc.2022.1024915 (PMC9691858; doi:10.3389/fonc.2022.1024915)

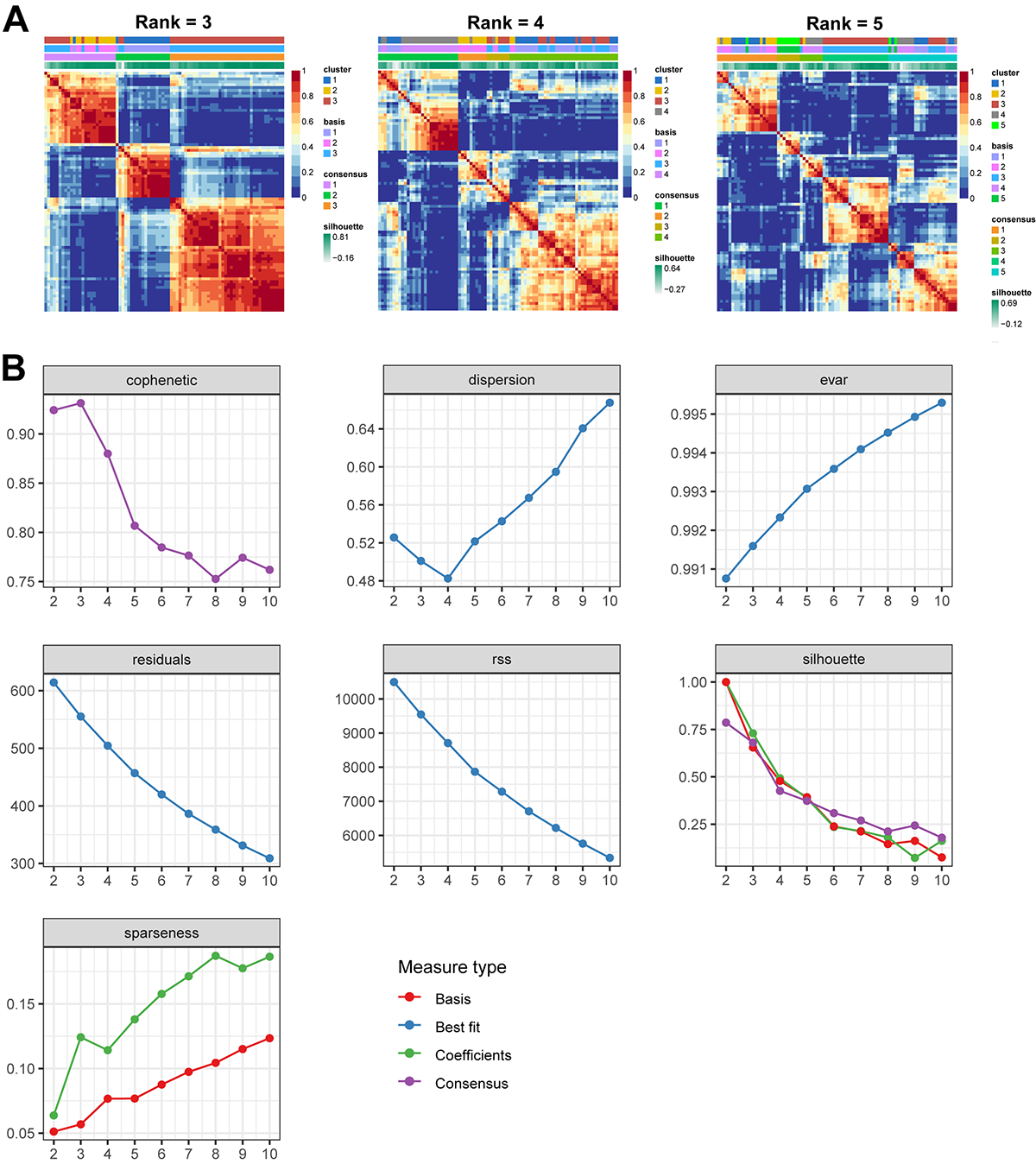

Supplement: Supplementary Figure 1 — Unsupervised consensus NMF clustering on TARGET cohort. (A) Heatmaps of NMF clustering for k = 3, 4, and 5. (B) The correlation among cophenetic, dispersion, evar, residuals, rss, silhouette and sparseness coefficients with reference to different cluster number. [file Image_1.tif]

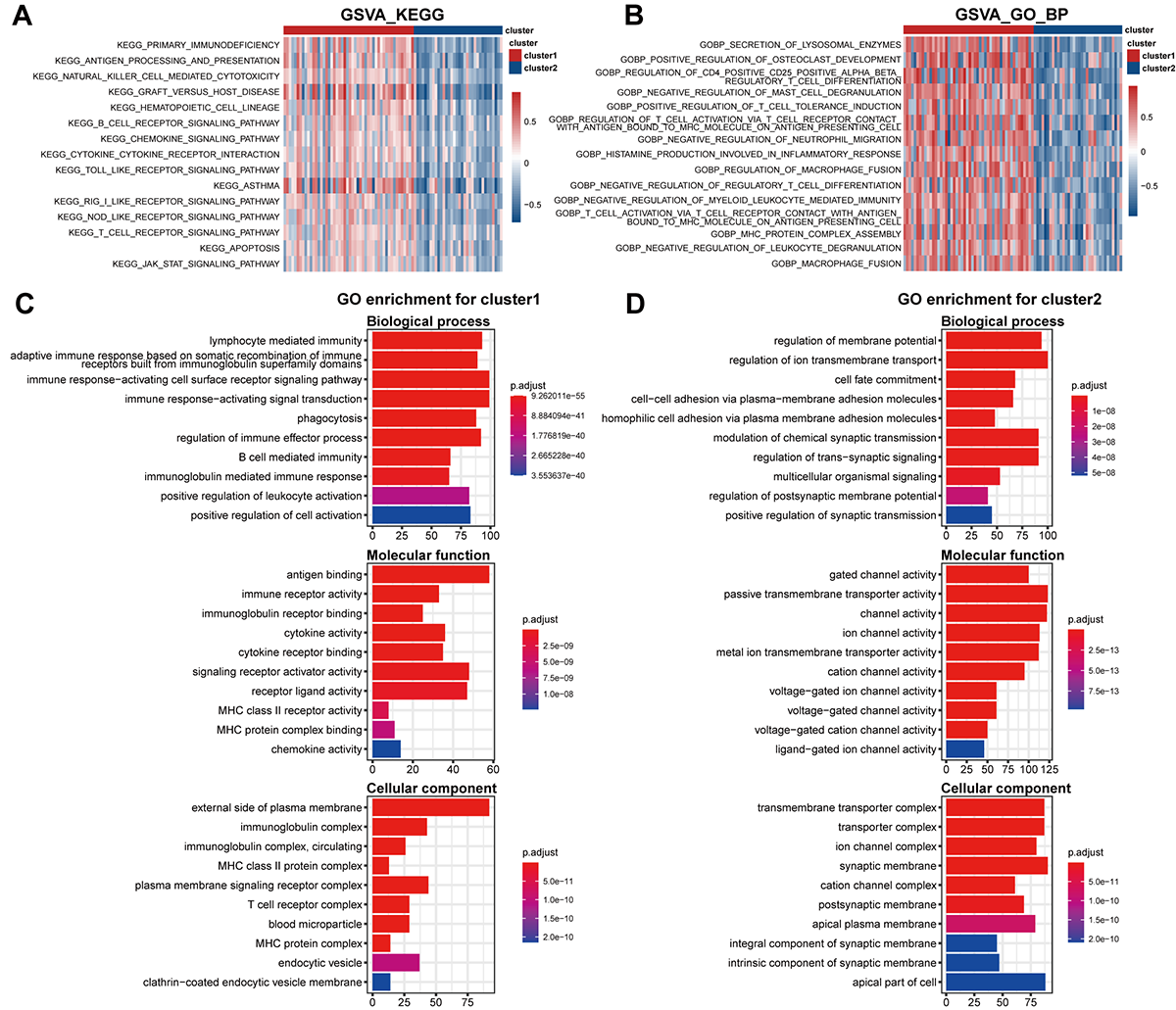

Supplement: Supplementary Figure 2 — Enrichment characteristics in two ferroptosis subclusters. (A, B) GSVA analysis for NMF clustered ferroptosis subclusters based on KEGG database and GO database. Red represents high expression level and blue represents low expression. The darker the color, the greater the significance. (C, D) GO enrichment analysis including biological process, molecular function and cellular component based on high-expression genes in cluster one and cluster two. The length of bars represents gene counts of GO terms. Color represents adjusted p value (Benjamini-Hochberg), the redder the color, the greater the significance. [file Image_2.tif]

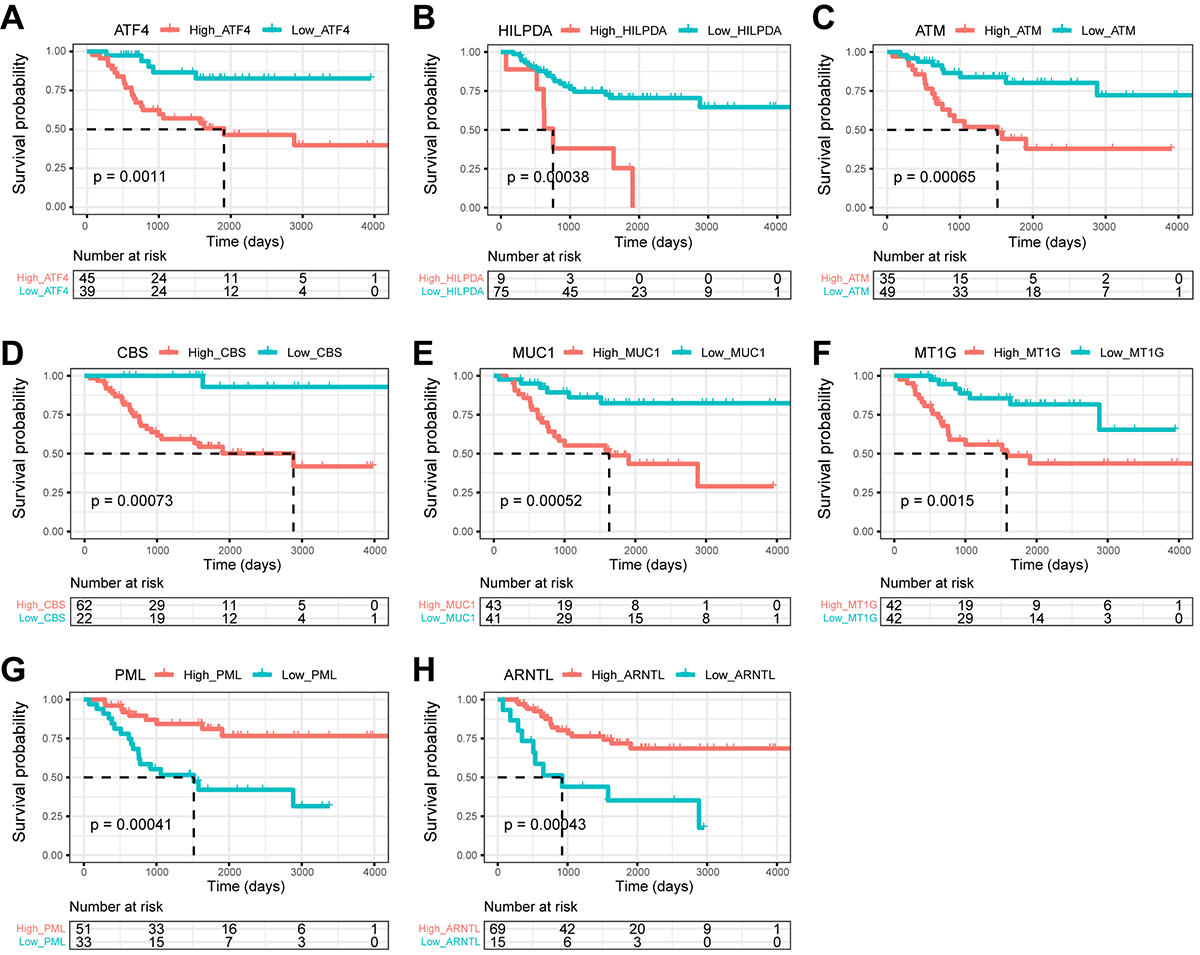

Supplement: Supplementary Figure 3 — Kaplan–Meier survival analysis for TARGET OS patients based on the expression of ATF4 (A), HILPDA (B), ATM (C), CBS (D), MUC1 (E), MT1G (F), PML (G), ARNTL (H). [file Image_3.tif]

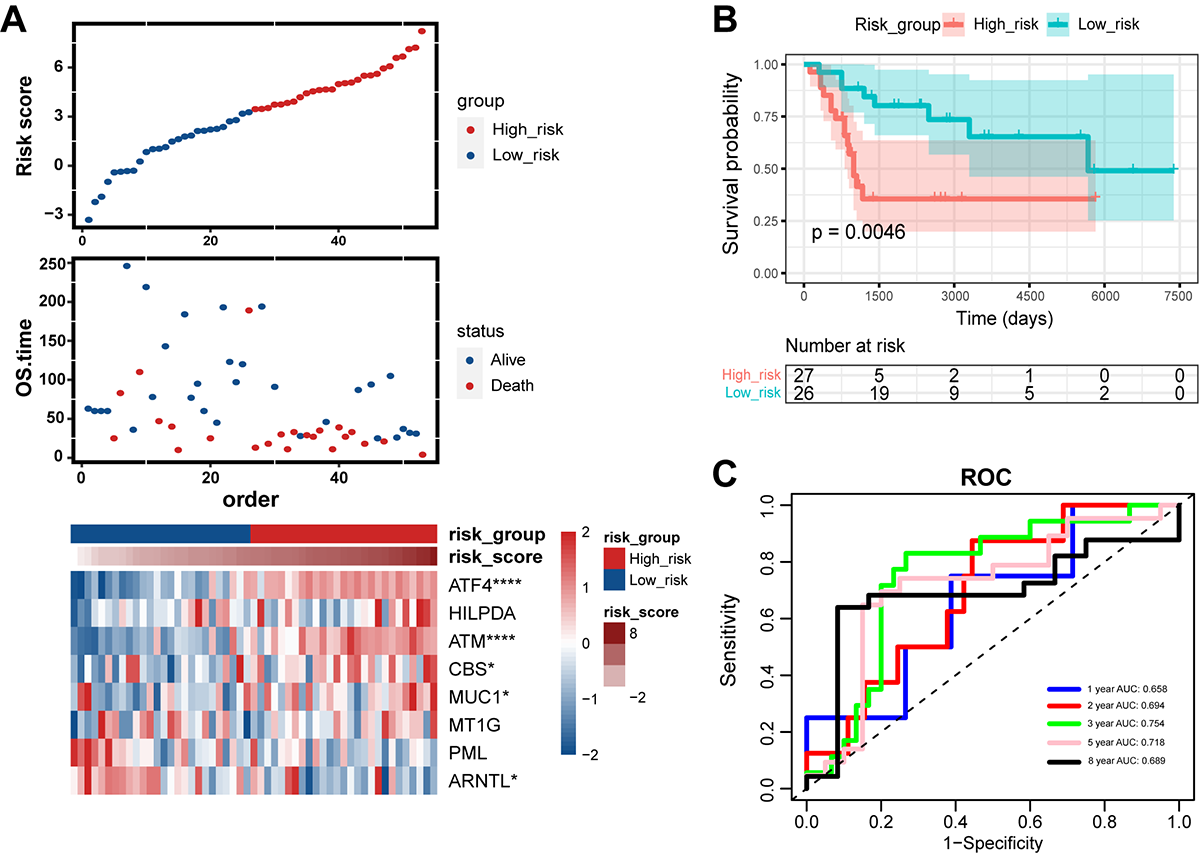

Supplement: Supplementary Figure 4 — Validation of the ferroptosis prognostic signature in GEO OS cohort. (A) Distribution plots of risk scores and heatmap of signature gene expression in GEO OS patients. Red represents high expression level and blue represents low expression. The darker the color, the greater the significance. (B) Kaplan-Meier analysis exhibiting the overall survival of GEO OS patients in high-risk group and low-risk group. (C) Time dependent ROC curve analysis of the ferroptosis signature model in predicting prognosis of GEO OS patients. * P < 0.05; ** P < 0.01; *** P < 0.001; **** P < 0.0001. [file Image_4.tif]

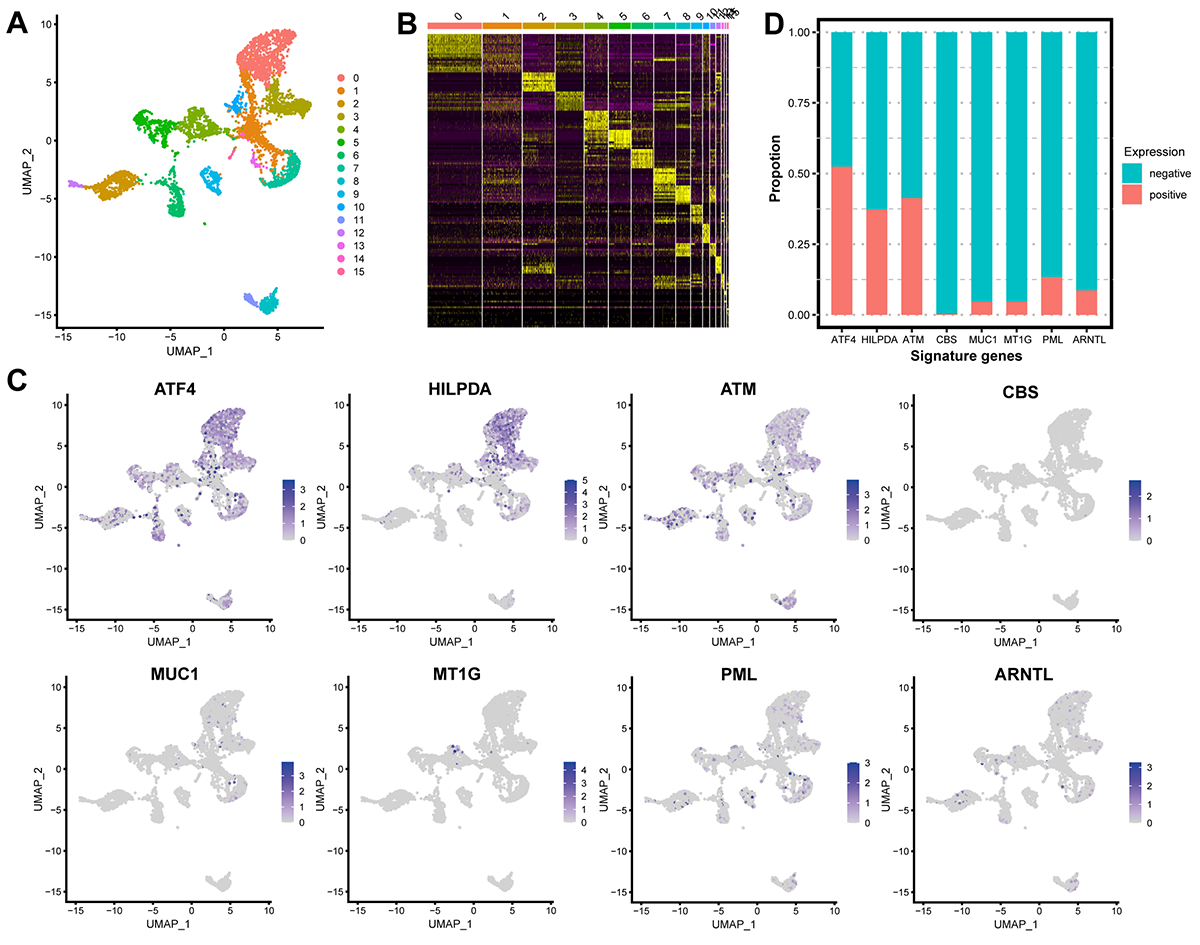

Supplement: Supplementary Figure 5 — Single cell sequencing analysis of primary OS samples. (A) UMAP visualized 16 cell subclusters identified using “FindClusters” function. (B) Heatmap of top10 feature genes in 16 subclusters. Yellow represents high expression level of genes, purple represents low expression. (C) Expression of eight risk signature genes in all identified cells. Purple represents high expression of signature genes. The more purple the color, the higher the expression. (D) Expression proportion of eight signature genes among all detected cells in primary OS samples. Red represents the proportion of gene-positive cells and blue represents the proportion of gene-negative cells. [file Image_5.tif]

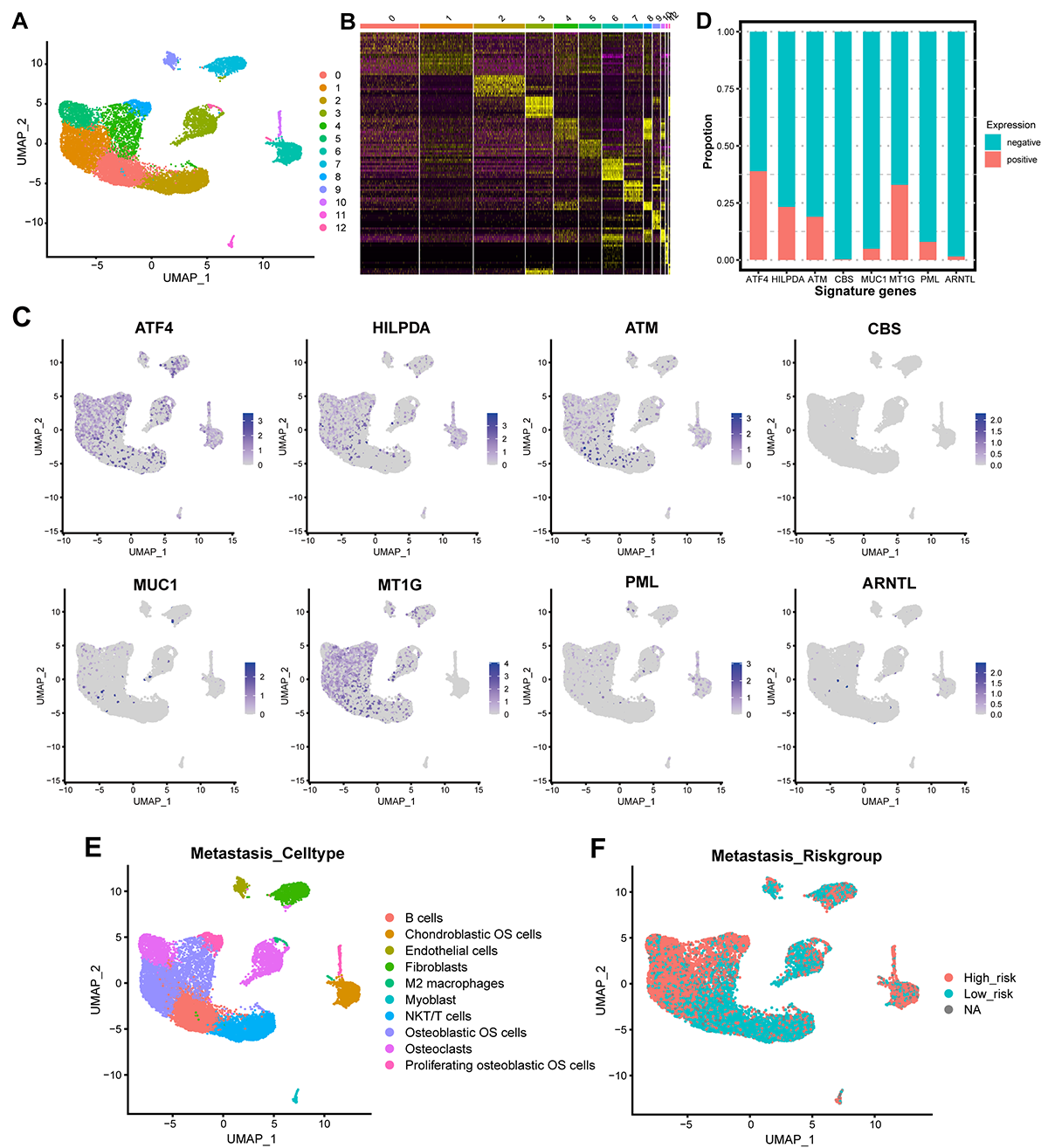

Supplement: Supplementary Figure 6 — Single cell sequencing analysis of metastatic OS samples. (A) UMAP visualized 13 cell subclusters identified in metastatic OS samples using “FindClusters” function. (B) Heatmap of top10 feature genes in the 13 subclusters. Yellow represents high expression level of genes, purple represents low expression. (C) Expression of eight risk signature genes in all identified cells. Purple represents high expression of signature genes. The more purple the color, the higher the expression. (D) Expression proportion of eight signature genes among all detected cells in metastatic OS samples. Red represents the proportion of gene-positive cells and blue represents the proportion of gene-negative cells. (E) UMAP visualization exhibits 10 annotated cell clusters based on metastatic OS single cell sequencing. (F) Risk cell clustering by ferroptosis signature clusters all cells into high-risk cells and low-risk cells. Red represents high-risk cells and blue represents low-risk cells. NA represents partial signature genes were not expressed in the single-cell sparse matrix. [file Image_6.tif]

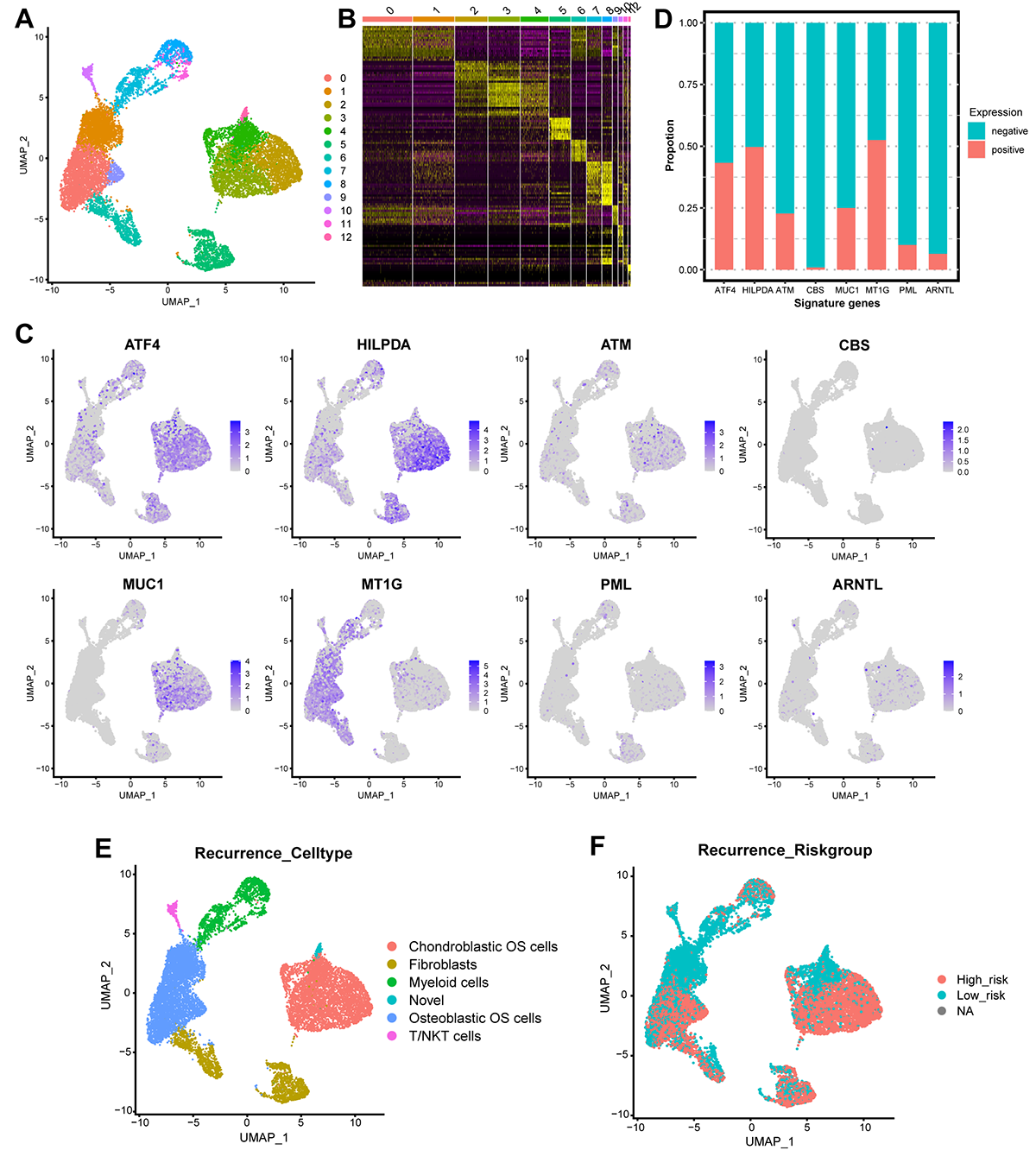

Supplement: Supplementary Figure 7 — Single cell sequencing analysis of recurrent OS samples. (A) UMAP visualized 13 cell subclusters identified in recurrent OS samples using “FindClusters” function. (B) Heatmap of top10 feature genes in the 13 subclusters. Yellow represents high expression level of genes; purple represents low expression. (C) Expression of eight risk signature genes in all identified cells. Purple represents high expression of signature genes. The more purple the color, the higher the expression. (D) Expression proportion of eight signature genes among all detected cells in recurrent OS samples. Red represents the proportion of gene-positive cells and blue represents the proportion of gene-negative cells. (E) UMAP visualization exhibits 6 annotated cell clusters based on recurrent OS single cell sequencing. (F) Risk cell clustering by ferroptosis signature clusters all cells into high-risk cells and low-risk cells. [file Image_7.tif]
