# Supplementary material for: A ferroptosis-related gene signature associated with immune landscape and therapeutic response in osteosarcoma
Source: Front Oncol. 2022 Nov 11;12:1024915. doi: 10.3389/fonc.2022.1024915 (PMC9691858; doi:10.3389/fonc.2022.1024915)
Supplement: Supplementary file 12 [file DataSheet_5.docx]

#### Target expression set matrix

#############

setwd("D:/WORK/bioinformatics/osteosarcoma/OS_Ferroptosis/TARGET")

Exp_T=read.table('TARGET-OS.htseq_counts.tsv.gz',na.strings = "NA",

sep='\t',quote=" ",fill=T,

comment.char = "!",header=T)

rownames(Exp_T)=Exp_T[,1]

annotation=read.table('gencode.v22.annotation.gene.probeMap',na.strings = "NA",

sep='\t',quote=" ",fill=T,

comment.char = "!",header=T)

a=annotation[,1:2]

b1=Exp_T[a$id,]

a$median=apply(b,1,median)

a1=a[order(a$median,decreasing = T),]

library(stringr)

a2=data.frame(id=a1$id,

ensembl_id=str_split(a1$gene,

'[.]',simplify = T)[,1],

median=a1$median)

a_nondup=a2[!duplicated(a2$ensembl_id),]

a_nondup1=a_nondup[a_nondup$median!=0,]

fix(a_nondup)

b1=b1[a_nondup1$id,]

rownames(b1)=a_nondup1$ensembl_id

b2=b1[,-1]

Exp_target=b2

save(Exp_target,file = "Exp_target.Rdata")

###clinical file

###survival file

########

library(data.table)

cli=fread("TARGET/TARGET-OS.clinical.tsv.gz",data.table = F)

surv=fread("TARGET/TARGET-OS.survival.tsv.gz",data.table = F)

rownames(surv)=surv$sample

cli=cli[-282,]

cli=cli[-270,]

cli=cli[-269,]

rownames(cli)=cli[,1]

sample_cli=cli$sample_id

library(stringr)

sample_cli=str_replace_all(sample_cli,c("-"="."))

cli1=cli

rownames(cli1)=sample_cli

cli=cli1

cli1=cli1[colnames(Exp_target),]

sample_surv=surv$sample

sample_surv=str_replace_all(sample_surv,c("-"="."))

rownames(surv)=sample_surv

surv=surv[colnames(Exp_target),]

save(cli,surv,Exp_target,file = "Exp_target.Rdata")

#####GEO data

######

#####21257

#####

rm(cli,Exp_target,surv)

library(GEOquery)

gset<-getGEO('GSE21257',destdir = ".",

AnnotGPL = F,

getGPL = F)

exprSet=exprs(gset[[1]])

gset[[1]]

pdata=pData(gset[[1]])

library(data.table)

setwd("D:/WORK/bioinformatics/osteosarcoma/OS_Ferroptosis/GSE21257")

annot=fread("GPL.txt",data.table = F)

a=annot[,-7]

a=a[,-(2:5)]

rownames(a)=a$ID

a=a[rownames(exprSet),]

a$median=apply(exprSet,1,median)

a=a[order(a$median,decreasing = T),]

a=a[!duplicated(a$Symbol),]

fix(a1)

a=a[a$Symbol!="",]

exprSet=exprSet[rownames(a),]

rownames(exprSet)=a$Symbol

boxplot(Exp_target,las=2)

cli_GEO21257=pdata

Exp_GEO21257=exprSet

setwd("D:/WORK/bioinformatics/osteosarcoma/OS_Ferroptosis/GEO")

save(cli_GSE21257,Exp_GSE21257,file = "GSE21257.Rdata")

cli_GSE21257=cli_GEO21257

Exp_GSE21257=Exp_GEO21257

##dat=log2(dat+1)

dat_exp=2^Exp_target-1

cli1=cli[-(3:4),]

fix(cli1)

surv=surv[-(3:4),]

fix(surv1)

surv1=surv[-46,]

surv1=surv1[-35,]

cli1=cli1[-46,]

cli1=cli1[-35,]

all(rownames(cli1)==rownames(surv1))

cli=cli1

surv=surv1

dat_exp=dat_exp[,rownames(cli)]

metadata=data.frame(ID=rownames(cli),sample=cli$`Disease at diagnosis`)

library(stringr)

write.csv(metadata,file = "metadata.csv")

library(data.table)

metadata=fread("metadata.csv",data.table = F)

metadata=metadata[,-1]

colnames(metadata)=metadata[1,]

metadata=metadata[-1,]

rownames(metadata)=metadata$ID

#sample <- ifelse(substring(metadata$ID,14,15)=="01","cancer","recur")

####

exprset=dat_exp

exprset=round(exprset,digits = 0)

exprssion=exprset[apply(exprset,1,function(x) sum(x>0)>=ncol(exprset)/2),]

group_list=dat$risk_group

tmp=data.frame(group=group_list)

###coldata

table(group_list)

rownames(tmp)=colnames(exprset)

library(DESeq2)

colData <- tmp

dds <- DESeqDataSetFromMatrix(countData = exprssion,

colData = colData,

design = ~ group)

dds <- DESeq(dds)

save(dds,file="scoregroup_dds.rdata")

rld=vst(dds,blind = F)

DEseq_norm=assay(rld)

save(DEseq_norm,DEG1,file = "scoregroup.rdata")

res1=results(dds,contrast = c("group","High_risk","Low_risk"))

resOrdered1<-res1[order(res1$padj),]

DEG1=as.data.frame(resOrdered1)

genes=DEG1[rownames(DEG1)%in% gene,]

####DEseq2

####

dat=round(dat,digits = 0)###integer

BiocManager::install('DESeq2')

library(DESeq2)

dds <-DESeqDataSetFromMatrix(countData=dat,

colData=metadata,

design=~sample)

dds <- DESeq(dds)

length(table(unique(rownames(dat))))

rld=vst(dds,blind = F)

DEseq_norm=assay(rld)#####DEseq_norm

save(dds,DEseq_norm,cli,surv,file="TARGET_DESeq2_DEseq_norm.rdata")

####

####ferroptosis/driver&suppressor

library(data.table)

ferr_genes=fread("ferroptosis_genes.csv",data.table = F)

ferr_genes=ferr_genes[!duplicated(ferr_genes$Symbol),]

rownames(ferr_genes)=ferr_genes$Symbol

ferr_g=ferr_genes[ferr_genes$other!="marker",]

ferr_Exp=DEseq_norm[rownames(ferr_g),]

DEseq_norm=as.data.frame(DEseq_norm)

ferr_Exp=na.omit(ferr_Exp)

setwd("D:/WORK/bioinformatics/osteosarcoma/OS_Ferroptosis/TARGET")

save(ferr_Exp,ferr_genes,ferr_g,file="ferr_genes.Rdata")

ag=sort(apply(ferr_Exp, 1, mad))

ag=as.data.frame(ag)

ag$ID=rownames(ag)

ag1=ag[ag$ag>0.5,]

data_f=ferr_Exp[rownames(ag1),]

data_f=na.omit(data_f)

data_scaled=t(scale(t(data_f)))

data_f = sweep(data_f,1, apply(data_f,1,median,na.rm=T))

head(data_f)[1:5,1:5]

library(ConsensusClusterPlus)

data_f=as.matrix(data_f)

results = ConsensusClusterPlus(data_f,maxK=5,reps=1000,pItem=0.8,

pFeature=1,clusterAlg="km",

title="median_k_e",

distance="euclidean",seed=1262118388.71279,

plot="pdf",

writeTable = T)

setwd("D:/WORK/bioinformatics/osteosarcoma/OS_Ferroptosis/TARGET/clusters/NMF")

library(NMF)

data_f=ferr_Exp[rownames(ag1),]

coad.log2fpkm.enengy <- data_f##

ranks <- 2:10

estim.coad <- nmf(coad.log2fpkm.enengy,ranks, nrun=50)

duplicated(colnames(coad.log2fpkm.enengy))

#Estimation of the rank: Quality measures computed from 10 runs for each value of r.

plot(estim.coad)

seed = 2020820

nmf.rank5 <- nmf(coad.log2fpkm.enengy,

rank = 5,

nrun=50,

seed = seed,

method = "brunet")

jco <- c("#2874C5","#EABF00","#C6524A","#868686","green")

index <- extractFeatures(nmf.rank5,"max")

sig.order <- unlist(index)

NMF.Exp.rank5 <- coad.log2fpkm.enengy[sig.order,]

NMF.Exp.rank5 <- na.omit(NMF.Exp.rank5)

group <- predict(nmf.rank5)

table(group)

consensusmap(nmf.rank5,

labRow = NA,

labCol = NA,

annCol = data.frame("cluster"=group[colnames(NMF.Exp.rank5)]),

annColors = list(cluster=c("1"=jco[1],"2"=jco[2],

"3"=jco[3],"4"=jco[4],"5"=jco[5])))

group=as.data.frame(group)

setwd("D:/WORK/bioinformatics/osteosarcoma/OS_Ferroptosis/TARGET/clusters/NMF")

save(group,surv,nmf.rank2,NMF.Exp.rank2,data_f,file = "NMF.Rdata")

library("FactoMineR")

library("factoextra")

# The variable group_list (index = 54676) is removed

# before PCA analysis

library(stringr)

group$group=str_c("group",group$group)

dat.pca <- PCA(t(data_f), graph = FALSE)

a=fviz_pca_ind(dat.pca,

geom.ind = "point", # show points only (nbut not "text")

col.ind = group$group, # color by groups

# palette = c("#00AFBB", "#E7B800"),

#addEllipses = TRUE, # Concentration ellipses

legend.title = "Groups"

)

ggsave(file="pca.pdf",plot = a,height = 4,width = 5)

####survival

#####

all(rownames(surv)==rownames(group))

surv$group=group$group

setwd("D:/WORK/bioinformatics/osteosarcoma/OS_Ferroptosis/TARGET/clusters/NMF")

library(ggplot2)

library(survival)

library(survminer)

table(group$event)

fit=surv_fit(Surv(OS.time,OS)~group,data = surv)

ggsurvplot(fit, data = surv,

surv.median.line = "hv", # Add medians survival

# Change legends: title & labels

legend.title = "group",

legend.labs = c("group1", "group2"),

# Add p-value and tervals

pval = TRUE,

conf.int = TRUE,

# Add risk table

risk.table = TRUE,

tables.height = 0.2,

tables.theme = theme_cleantable(),

# Color palettes. Use custom color: c("#E7B800", "#2E9FDF"),

# or brewer color (e.g.: "Dark2"), or ggsci color (e.g.: "jco")

ggtheme = theme_bw() # Change ggplot2 theme

)

#####

#####DEG&heatmap

setwd("D:/WORK/bioinformatics/osteosarcoma/OS_Ferroptosis/TARGET/clusters/NMF")

group$ID=rownames(group)

group=group[order(group$group,decreasing = F),]

data_order=data_f[,rownames(group)]

library(limma)

group_list=group$group

design=model.matrix(~0+factor(group_list))

colnames(design)=c("group1","group2")

head(design)

rownames(design)=group$ID

deg = function(data_order,design,contrast.matrix){

##step1

fit <- lmFit(data_order,design)

##step2

fit2 <- contrasts.fit(fit, contrast.matrix)

fit2 <- eBayes(fit2) ## default no trend !!!

##eBayes() with trend=TRUE

##step3

tempOutput = topTable(fit2, coef=1, n=Inf)

nrDEG = na.omit(tempOutput)

#write.csv(nrDEG2,"limma_notrend.results.csv",quote = F)

head(nrDEG)

return(nrDEG)

}

####group2 VS group1

contrast.matrix=makeContrasts("group2-group1",levels=design)

deg1=deg(data_order,design,contrast.matrix)

deg1$name=rownames(deg1)

deg1=deg1[order(deg1$logFC,decreasing = T),]

data_order=data_order[rownames(deg1),]

library(pheatmap)

rownames(group)=colnames(data_order)

all(colnames(data_order)==rownames(group))

data_order=t(scale(t(data_order)))

data_order[data_order< -1.5]= -1.5

data_order[data_order>1.5]=1.5

table(group$group)

fix(group)

group$ID=1

pheatmap(data_order,show_colnames = F,show_rownames = T,border_color=NA,

cluster_rows = F,cluster_cols = F,annotation_col = group,

cellwidth=3,cellheight=2,color = colorRampPalette(c("purple", "black", "yellow"))(50))

pheatmap(data_order,show_colnames = F,show_rownames = T,border_color=NA,

cluster_rows = F,cluster_cols = F,annotation_col = group,

cellwidth=3.5,cellheight=2,color = colorRampPalette(c("#104E8B", "white", "#CD2626"))(50))

setwd("D:/WORK/bioinformatics/osteosarcoma/OS_Ferroptosis/TARGET/clusters/NMF")

save(deg1,data_order,group,file = "subgroup2_deg_ferr.Rdata")

save(data_f,group,surv,file = "after_cluster.Rdata")

#####

#####GSVA

#####GSVA

library(ggplot2)

library(clusterProfiler)

library(org.Hs.eg.db)

library(GSVA)

library(GSEABase)

library(pheatmap)

DEseq_norm=as.matrix(DEseq_norm)

geneSets <- getGmt('c2.cp.kegg.v7.4.symbols.gmt')

es.max <- gsva(DEseq_norm, geneSets,

mx.diff=FALSE, verbose=FALSE,

parallel.sz=1)

library(limma)

group_list=group$group

es.max=es.max[,rownames(group)]

design=model.matrix(~0+factor(group_list))

colnames(design)=c("group1","group2")

head(design)

rownames(design)=rownames(group)

deg = function(es.max,design,contrast.matrix){

##step1

fit <- lmFit(es.max,design)

##step2

fit2 <- contrasts.fit(fit, contrast.matrix)

fit2 <- eBayes(fit2) ## default no trend !!!

##eBayes() with trend=TRUE

##step3

tempOutput = topTable(fit2, coef=1, n=Inf)

nrDEG = na.omit(tempOutput)

#write.csv(nrDEG2,"limma_notrend.results.csv",quote = F)

head(nrDEG)

return(nrDEG)

}

####group2 VS group1

contrast.matrix=makeContrasts("group2-group1",levels=design)

deg_KEGG=deg(es.max,design,contrast.matrix)

#deg1=deg1[order(deg1$logFC,decreasing = T),]

deg_KEGG=deg_KEGG[deg_KEGG$P.Value<0.05,]

deg_KEGG$name=rownames(deg_KEGG)

geneset2 <- getGmt('c5.go.bp.v7.4.symbols.gmt')

es.max_bp <- gsva(DEseq_norm, geneset2,

mx.diff=FALSE, verbose=FALSE,

parallel.sz=1)

library(limma)

group_list=group$group

es.max_bp=es.max_bp[,rownames(group)]

design=model.matrix(~0+factor(group_list))

colnames(design)=c("group1","group2")

head(design)

rownames(design)=rownames(group)

deg = function(es.max_bp,design,contrast.matrix){

##step1

fit <- lmFit(es.max_bp,design)

##step2

fit2 <- contrasts.fit(fit, contrast.matrix)

fit2 <- eBayes(fit2) ## default no trend !!!

##eBayes() with trend=TRUE

##step3

tempOutput = topTable(fit2, coef=1, n=Inf)

nrDEG = na.omit(tempOutput)

#write.csv(nrDEG2,"limma_notrend.results.csv",quote = F)

head(nrDEG)

return(nrDEG)

}

####group2 VS group1

contrast.matrix=makeContrasts("group2-group1",levels=design)

deg_GO_BP=deg(es.max_bp,design,contrast.matrix)

#deg1=deg1[order(deg1$logFC,decreasing = T),]

deg_GO_BP=deg_GO_BP[deg_GO_BP$P.Value<0.05,]

deg_GO_BP$name=rownames(deg_GO_BP)

setwd("D:/WORK/bioinformatics/osteosarcoma/OS_Ferroptosis/TARGET/clusters/NMF")

write.csv(deg_KEGG,file = "deg_KEGG.csv")

write.csv(deg_GO_BP,file="deg_GO.csv")

deg_KEGG_note=read.csv("deg_KEGG.csv")

es.max=es.max[deg_KEGG_note$name,]

es.max[es.max< -1]= -1

es.max[es.max>1]=1

ann_colors = list(

ID = "white",

group = c(group1 = "#CD2626", group2 = "#104E8B")

)

pheatmap(es.max,show_colnames = F,show_rownames = T,border_color=NA,

cluster_rows = F,cluster_cols = F,annotation_col = group,

cellwidth=3,cellheight=18,

annotation_colors = ann_colors,

color = colorRampPalette(c("#104E8B", "white", "#CD2626"))(50))

deg_GO_note=read.csv("deg_GO.csv")

es.max_bp=es.max_bp[deg_GO_note$name,]

es.max_bp[es.max_bp< -1]= -1

es.max_bp[es.max_bp>1]=1

pheatmap(es.max_bp,show_colnames = F,show_rownames = T,border_color=NA,

cluster_rows = F,cluster_cols = F,annotation_col = group,

cellwidth=3,cellheight=18,annotation_colors = ann_colors,

color = colorRampPalette(c("#104E8B", "white", "#CD2626"))(50))

save(deg_KEGG,deg_GO_BP,DEseq_norm,group,surv,file="GSVA.Rdata")

######enrichment

#####

C2=deg2[deg2$logFC>0.5,]

C1=deg2[deg2$logFC< -0.5,]

library(clusterProfiler)

library(enrichplot)

library(DOSE)

library(org.Hs.eg.db)

gene2 <- rownames(C2)

gene2 = bitr(gene2, fromType="SYMBOL", toType="ENTREZID", OrgDb="org.Hs.eg.db")

de2 <- gene2$ENTREZID

go2 <- enrichGO(gene = de2, OrgDb = "org.Hs.eg.db", ont="all")

library(ggplot2)

p2 <- dotplot(go2, split="ONTOLOGY",title="GO_C2") +facet_grid(ONTOLOGY~., scale="free")

p2

C2_enrich=go2@result

gene1 <- rownames(C1)

gene1 = bitr(gene1, fromType="SYMBOL", toType="ENTREZID", OrgDb="org.Hs.eg.db")

de1 <- gene1$ENTREZID

go1 <- enrichGO(gene = de1, OrgDb = "org.Hs.eg.db", ont="all")

library(ggplot2)

p1 <- dotplot(go1, split="ONTOLOGY",title="GO_C1") +facet_grid(ONTOLOGY~., scale="free")

p1

C1_enrich=go1@result

setwd("D:/WORK/bioinformatics/OA_Ferroptosis/clusters_for_ferr/figs")

write.csv(C1_enrich,file = "C1_enrich.csv")

write.csv(C2_enrich,file = "C2_enrich.csv")

kk1=enrichKEGG(gene=de1,

organism='hsa',

pvalueCutoff=0.05,

qvalueCutoff=0.05)

kegg1 <- setReadable(kk1, OrgDb=org.Hs.eg.db, keyType="ENTREZID")

kk2=enrichKEGG(gene=de2,

organism='hsa',

pvalueCutoff=0.1,

qvalueCutoff=0.1)

kegg2 <- setReadable(kk2, OrgDb=org.Hs.eg.db, keyType="ENTREZID")

dotplot(kk1,title="KEGG_C1",showCategory=20)

dotplot(kk2,title="KEGG_C2",showCategory=20)

C1_KEGG=kegg1@result

C2_KEGG=kegg2@result

write.csv(C1_KEGG,file = "C1_KEGG.csv")

write.csv(C2_KEGG,file = "C2_KEGG.csv")

####

####ssGSEA

library(genefilter)

library(GSVA)

library(Biobase)

gsva_matrix=gsva(DEseq_norm,list,method="ssgsea",

kcdf="Gaussian",abs.ranking=T)

save(gsva_matrix,file = "step9_ssGSEA.rdata")

library(limma)

group_list=group$group

gsva_matrix=gsva_matrix[,rownames(group)]

design=model.matrix(~0+factor(group_list))

colnames(design)=c("group1","group2")

head(design)

rownames(design)=rownames(group)

deg = function(gsva_matrix,design,contrast.matrix){

##step1

fit <- lmFit(gsva_matrix,design)

##step2

fit2 <- contrasts.fit(fit, contrast.matrix)

fit2 <- eBayes(fit2) ## default no trend !!!

##eBayes() with trend=TRUE

##step3

tempOutput = topTable(fit2, coef=1, n=Inf)

nrDEG = na.omit(tempOutput)

#write.csv(nrDEG2,"limma_notrend.results.csv",quote = F)

head(nrDEG)

return(nrDEG)

}

####group2 VS group1

contrast.matrix=makeContrasts("group2-group1",levels=design)

deg1=deg(gsva_matrix,design,contrast.matrix)

setwd("D:/WORK/bioinformatics/osteosarcoma/OS_Ferroptosis/TARGET/clusters/NMF")

save(gsva_matrix,group,deg1,file = "ssGSEA.rdata")

matrix=data.frame(group=rep(group_list,28),

cell=unlist(lapply(1:28,function(i) rep(rownames(gsva_matrix)[i],84))),

value=unlist(lapply(1:28, function(i) gsva_matrix[i,])))

library(ggpubr)

ggboxplot(matrix,x="cell",y="value",fill="group",

xlab="",

ylab="ssGSEA score of each immune cell",

title="association between different groups and immune cells",

x.text.angle=45)+stat_compare_means(aes(x=cell,y=value,fill=group),method="kruskal.test",label = "p.signif")

####

####GSVA

library(ggplot2)

library(clusterProfiler)

library(org.Hs.eg.db)

library(GSVA)

library(GSEABase)

library(pheatmap)

DEseq_norm=as.matrix(DEseq_norm)

setwd("D:/WORK/bioinformatics/osteosarcoma/OS_Ferroptosis/TARGET/clusters/NMF")

geneSets <- getGmt('c2.cp.kegg.v7.4.symbols.gmt')

es.max <- gsva(DEseq_norm, geneSets,

mx.diff=FALSE, verbose=FALSE,

parallel.sz=1)

library(limma)

group_list=dat$risk_group

es.max=es.max[,rownames(dat)]

design=model.matrix(~0+factor(group_list))

colnames(design)=c("High_risk","Low_risk")

head(design)

rownames(design)=rownames(dat)

deg = function(es.max,design,contrast.matrix){

##step1

fit <- lmFit(es.max,design)

##step2

fit2 <- contrasts.fit(fit, contrast.matrix)

fit2 <- eBayes(fit2) ## default no trend !!!

##eBayes() with trend=TRUE

##step3

tempOutput = topTable(fit2, coef=1, n=Inf)

nrDEG = na.omit(tempOutput)

#write.csv(nrDEG2,"limma_notrend.results.csv",quote = F)

head(nrDEG)

return(nrDEG)

}

####group2 VS group1

contrast.matrix=makeContrasts("Low_risk-High_risk",levels=design)

deg_KEGG=deg(es.max,design,contrast.matrix)

#deg1=deg1[order(deg1$logFC,decreasing = T),]

deg_KEGG=deg_KEGG[deg_KEGG$P.Value<0.05,]

deg_KEGG$name=rownames(deg_KEGG)

geneset2 <- getGmt('c5.go.bp.v7.4.symbols.gmt')

es.max_bp <- gsva(DEseq_norm, geneset2,

mx.diff=FALSE, verbose=FALSE,

parallel.sz=1)

deg = function(es.max_bp,design,contrast.matrix){

##step1

fit <- lmFit(es.max_bp,design)

##step2

fit2 <- contrasts.fit(fit, contrast.matrix)

fit2 <- eBayes(fit2) ## default no trend !!!

##eBayes() with trend=TRUE

##step3

tempOutput = topTable(fit2, coef=1, n=Inf)

nrDEG = na.omit(tempOutput)

#write.csv(nrDEG2,"limma_notrend.results.csv",quote = F)

head(nrDEG)

return(nrDEG)

}

####group2 VS group1

deg_GO_BP=deg(es.max_bp,design,contrast.matrix)

#deg1=deg1[order(deg1$logFC,decreasing = T),]

deg_GO_BP=deg_GO_BP[deg_GO_BP$P.Value<0.05,]

deg_GO_BP$name=rownames(deg_GO_BP)

setwd("D:/WORK/bioinformatics/osteosarcoma/OS_Ferroptosis/TARGET/clusters/NMF")

write.csv(deg_KEGG,file = "GSVA_KEGG_riskgroups.csv")

write.csv(deg_GO_BP,file="GSVA_GO_riskgroups.csv")

deg_KEGG_note=read.csv("deg_KEGG.csv")

es.max=es.max[deg_KEGG_note$name,]

es.max[es.max< -1]= -1

es.max[es.max>1]=1

ann_colors = list(

ID = "white",

group = c(group1 = "#CD2626", group2 = "#104E8B")

)

pheatmap(es.max,show_colnames = F,show_rownames = T,border_color=NA,

cluster_rows = F,cluster_cols = F,annotation_col = group,

cellwidth=3,cellheight=18,

annotation_colors = ann_colors,

color = colorRampPalette(c("#104E8B", "white", "#CD2626"))(50))

deg_GO_note=read.csv("deg_GO.csv")

es.max_bp=es.max_bp[deg_GO_note$name,]

es.max_bp[es.max_bp< -1]= -1

es.max_bp[es.max_bp>1]=1

pheatmap(es.max_bp,show_colnames = F,show_rownames = T,border_color=NA,

cluster_rows = F,cluster_cols = F,annotation_col = group,

cellwidth=3,cellheight=18,annotation_colors = ann_colors,

color = colorRampPalette(c("#104E8B", "white", "#CD2626"))(50))

save(deg_KEGG,deg_GO_BP,DEseq_norm,group,surv,file="GSVA.Rdata")

####

####ESTIMATE

options(stringsAsFactors = F)

library(utils)

library(utils)

rforge<-"http://r-forge.r-project.org"

install.packages("estimate",repos=rforge,dependencies=TRUE)

library(estimate)

dat=fp

estimate <- function(dat,pro){

input.f=paste0(pro,'_estimate_input.txt')

output.f=paste0(pro,'_estimate_gene.gct')

output.ds=paste0(pro,'_estimate_score.gct')

write.table(dat,file = input.f,sep = '\t',quote = F)

library(estimate)

filterCommonGenes(input.f=input.f,

output.f=output.f ,

id="GeneSymbol")

estimateScore(input.ds = output.f,

output.ds=output.ds,

platform="affymetrix")

scores=read.table(output.ds,skip = 2,header = T)

rownames(scores)=scores[,1]

scores=t(scores[,3:ncol(scores)])

return(scores)

}

pro='OS'

scores1=estimate(DEseq_norm,pro)

rownames(scores1)=colnames(DEseq_norm)

dat$group=ifelse(dat$fp>median(dat$fp),"high","low")

grouplist=dat$risk_group

scores1=scores1[rownames(dat),]

scores1=scale(scores1)

es.max1=t(scores1)

matrix=data.frame(group=rep(grouplist,4),

cell=unlist(lapply(1:4,function(i) rep(rownames(es.max1)[i],84))),

value=unlist(lapply(1:4, function(i) es.max1[i,])))

library(ggpubr)

ggboxplot(matrix,x="cell",y="value",fill="group",

xlab="",

ylab="Scaled ESTIMATE scores",

title="Association between ESTIMATE scores and risk groups",

x.text.angle=45,

legend.labs = c("high", "low"))+

stat_compare_means(aes(x=cell,y=value,fill=group),

method="t.test",label = "p.format")#+

#geom_jitter(aes(fill=group),width =0.2,shape = 21,size=2)

fp1=fp[colnames(es.max1),]

es.max1=t(es.max1)

all(rownames(es.max1)==rownames(fp1))

es.max=cbind(es.max1,fp1)

mytheme=theme(plot.title = element_text(face = "bold",size = "12",color = "black",hjust = 0.5),

axis.title = element_text(face = "bold",size = "10",color = "black"),

axis.text = element_text(face = "bold",size = "9",color = "black"),

axis.line = element_line(size = 1),

panel.grid.major.y = element_line(color = "grey",linetype = 1),

panel.grid.minor.y = element_line(color = "grey",linetype = 2),

panel.background = element_rect(fill = "white",color = "black",size=1.5),

panel.grid.major = element_blank()+

scale_y_continuous(breaks = -1,-0.5,0,0.5,1,1.5))

ggplot(data=es.max,aes(x=fp,y=TumorPurity))+

geom_point(color="black",fill="black",size=4)+

geom_smooth(method = "lm",se=F)+

labs(title = "Correlation between Risk score and TumorPurity",Y = "TumorPurity")+

mytheme

cor_risk_TP=cor(es.max$fp,es.max$TumorPurity,method = "spearman")

cor_risk_S=cor(es.max$fp,es.max$StromalScore,method = "spearman")

cor_risk_I=cor(es.max$fp,es.max$ImmuneScore,method = "spearman")

cor_risk_E=cor(es.max$fp,es.max$ESTIMATEScore,method = "spearman")

es.max=es.max[,-6]

library(Hmisc)

res2<-rcorr(as.matrix(es.max),type = "spearman")

res2$r

res2$P

save(es.max,cor_risk_E,cor_risk_I,cor_risk_S,cor_risk_TP,file = "ESTIMATE_risk.Rdata")

####

####COX

library("survival")

genelist1=genelist[-RP5-857K21]

univ_formulas <- sapply(genelist,

function(x) as.formula(paste('Surv(OS.time,OS)~', x)))

univ_models <- lapply( univ_formulas, function(x){coxph(x, data = data)})

univ_results <- lapply(univ_models,

function(x){

x <- summary(x)

p.value<-signif(x$wald["pvalue"], digits=2)

HR <-signif(x$coef[2], digits=2);

HR.confint.lower <- signif(x$conf.int[,"lower .95"], 2)

HR.confint.upper <- signif(x$conf.int[,"upper .95"],2)

HR <- paste0(HR, " (",

HR.confint.lower, "-", HR.confint.upper, ")")

res<-c(p.value,HR)

names(res)<-c("p.value","HR (95% CI for HR)")

return(res)

})

res <- t(as.data.frame(univ_results, check.names = FALSE))

as.data.frame(res)

res=as.data.frame(res)

respvalue=res[res$p.value<0.05,]

setwd("D:/WORK/bioinformatics/osteosarcoma/OS_Ferroptosis/TARGET/clusters/NMF/Rdata")

save(res,respvalue,surv,data,data_f,group,DEseq_norm,file = "COX.Rdata")

####LASSO

####

library(dplyr)

library(ggplot2)

library(survival)

BiocManager::install("lars")

library(lars)

BiocManager::install("glmSparseNet")

library(glmSparseNet)

data1=data1[,-1]

data=data[,rownames(respvalue)]

data1=data1[,rownames(respvalue)]

data1=as.matrix(data1)

cv_fit <- cv.glmnet(x=data1, y=Surv(surv$OS.time,surv$OS),

alpha = 1, family ="cox")

fit<- glmnet(x=data1, y=Surv(surv$OS.time,surv$OS), alpha = 1, family ="cox")

plot(cv_fit)

plot(fit, xvar = "norm", label = TRUE)

c(cv_fit$lambda.min,cv_fit$lambda.1se)

fit<- glmnet(x=data1, y=Surv(surv$OS.time,surv$OS), alpha = 1, lambda=cv_fit$lambda.min,family = "cox" )

choose_gene=rownames(fit$beta)[as.numeric(fit$beta)!=0]

choose_gene

save(choose_gene,surv,data,data1,DEseq_norm,group,file = "LASSO.Rdata")

####multi-COX

####

multicoxdat=data1[,choose_gene]

rownames(multicoxdat)==rownames(surv)

mcoxdat=cbind(surv,multicoxdat)

res.cox <- coxph(Surv(OS.time, OS) ~

ARNTL + PML + ATF4+G6PD+ATM+HILPDA+MUC1+SCD+FADS2+CBS+MT1G,

data = mcoxdat)

tstep<-step(res.cox)

summary(tstep)

res.cox1 <- coxph(Surv(OS.time, OS) ~

ARNTL + PML + ATF4 + ATM +

HILPDA + MUC1 + CBS + MT1G,

data = mcoxdat)

x <- summary(res.cox1)

pvalue=signif(as.matrix(x$coefficients)[,5],2)

HR=signif(as.matrix(x$coefficients)[,2],2)

low=signif(x$conf.int[,3],2)

high=signif(x$conf.int[,4],2)

multi_res=data.frame(p.value=pvalue,

HR=paste(HR," (",low,"-",high,")",sep=""),

stringsAsFactors = F

)

multi_res

save(res.cox1,mcoxdat,multi_res,file = "multiCOX.Rdata")

fp=predict(res.cox1,mcoxdat,type="risk")

fp=as.data.frame(fp)

save(fp,file = "risk_score.Rdata")

#####

#####forrest_visualization

#####

setwd("D:/WORK/bioinformatics/osteosarcoma/OS_Ferroptosis/TARGET/clusters/NMF")

write.csv(multi_res,file = "multicox_res.csv")

write.csv(respvalue,file = "cox_res.csv")

multicox_res=read.csv("multicox_res.csv")

multicox_res=multicox_res[order(multicox_res$HR,decreasing = T),]

cox_res=cox_res[order(cox_res$HR,decreasing = T),]

rownames(multicox_res)=multicox_res$gene.symbol

cox_res=read.csv("cox_res.csv")

cox_res=cox_res[order(cox_res$HR,decreasing = T),]

rownames(cox_res)=cox_res$gene.symbol

save(multicox_res,file = "multiCOX.Rdata")

save(res,cox_res,surv,data,data_f,group,DEseq_norm,file = "COX.Rdata")

cox_res<-data.frame(cox_res,stringsAsFactors=FALSE)

cox_res[,2]<-as.numeric(cox_res[,2])

cox_res[,5]<-as.numeric(cox_res[,5])

cox_res[,6]<-as.numeric(cox_res[,6])

cox_res[,7]<-as.numeric(cox_res[,7])

cox_res=cox_res[order(cox_res$HR,decreasing = F),]

cox_res$gene.symbol=factor(cox_res$gene.symbol,order=TRUE,

levels =c(cox_res$gene.symbol))

library(ggplot2)

ggplot(cox_res,aes(x=HR,y=gene.symbol,color=P.value))+

geom_errorbarh(aes(xmax=High.95.CI,xmin=Low.95.CI),color="black",

height=0,size=0.8)+

geom_point(aes(x=HR,y=gene.symbol),size=4,shape=18)+

geom_vline(xintercept=1,linetype="dashed",size=1.2)+

scale_x_continuous(breaks=c(0,0.5,1,1.5,2,2.5,3.0))+

scale_y_discrete(labels=c(as.character(cox_res[,1])))+

scale_color_continuous(low="dodgerblue4",high="lightskyblue")+

ylab("Gene")+xlab("Hazard ratios")+

coord_trans(x="log2")+

labs(color="P value",title="")+

theme(panel.grid.major =element_blank(), panel.grid.minor = element_blank(),panel.background = element_blank(),axis.line = element_line(colour = "black"))+

theme(axis.text=element_text(colour="black",size=14))+

theme(legend.text=element_text(size=14))+

theme(legend.title=element_text(size=15))+

theme(axis.title=element_text(colour="black",size=14))

library("timeROC")

library("survival")

with(dat,

ROC<<-timeROC(T=OS.time,

delta=OS,

marker=fp,

cause=1,

weighting="marginal",

times=c(365,730,1095,1460,1825),

ROC=TRUE,

iid=TRUE)

)

plot(ROC,time=365,col="blue",add=FALSE)

plot(ROC,time=730,col="red",add=T)

plot(ROC,time=1095,col="green",add=T)

plot(ROC,time=1825,col="pink",add=T)

ROC$AUC

####

####scRNA

library(SingleCellExperiment)

library(Seurat)

library(tidyverse)

library(Matrix)

library(scales)

library(cowplot)

library(RCurl)

sce2 <- CreateSeuratObject(Read10X('D:/WORK/bioinformatics/osteosarcoma/scRNA/BC2'),

min.features = 100)

sce3 <- CreateSeuratObject(Read10X('D:/WORK/bioinformatics/osteosarcoma/scRNA/BC3'),

min.features = 100)

sce5 <- CreateSeuratObject(Read10X('D:/WORK/bioinformatics/osteosarcoma/scRNA/BC5'),

min.features = 100)

sce6 <- CreateSeuratObject(Read10X('D:/WORK/bioinformatics/osteosarcoma/scRNA/BC6'),

min.features = 100)

sce10 <- CreateSeuratObject(Read10X('D:/WORK/bioinformatics/osteosarcoma/scRNA/BC10'),

min.features = 100)

sce11 <- CreateSeuratObject(Read10X('D:/WORK/bioinformatics/osteosarcoma/scRNA/BC11'),

min.features = 100)

sce16 <- CreateSeuratObject(Read10X('D:/WORK/bioinformatics/osteosarcoma/scRNA/BC16'),

min.features = 100)

sce17 <- CreateSeuratObject(Read10X('D:/WORK/bioinformatics/osteosarcoma/scRNA/BC17'),

min.features = 100)

sce20 <- CreateSeuratObject(Read10X('D:/WORK/bioinformatics/osteosarcoma/scRNA/BC20'),

min.features = 100)

sce21 <- CreateSeuratObject(Read10X('D:/WORK/bioinformatics/osteosarcoma/scRNA/BC21'),

min.features = 100)

sce22 <- CreateSeuratObject(Read10X('D:/WORK/bioinformatics/osteosarcoma/scRNA/BC22'),

min.features = 100)

save(sce2,sce3,sce5,sce6,sce10,sce11,sce16,sce17,sce20,sce21,sce22,

file = "sce_of_11_patient.Rdata")

merged_seurat2_3 <- merge(x = sce2,

y = sce3,

add.cell.id = c("BC2", "BC3"))

head(merged_seurat16_2_@meta.data)

tail(merged_seurat16_2_@meta.data)

merged_seurat5_6 <- merge(x = sce5,

y = sce6,

add.cell.id = c("BC5", "BC6"))

merged_seurat21_22 <- merge(x = sce21,

y = sce22,

add.cell.id = c("BC21", "BC22"))

merged_seurat16_2_ <- merge(x = sce16,

y = merged_seurat21_22,

add.cell.id = c("BC16"," "))

merged_seurat2_6<- merge(x = merged_seurat2_3,

y = merged_seurat5_6)

merged_seurat<- merge(x = seurat_primary,

y = merged_seurat16_2_)

seurat_primary=merged_seurat21_22

seurat_primary522=merged_seurat5_22

setwd("D:/WORK/bioinformatics/osteosarcoma/scRNA/primary")

save(seurat_primary,file = "seurat_primary.Rdata")

save(seurat_primary522,file = "seurat_primary522.Rdata")

seurat_meta <- merge(x = sce10,

y = sce17,

add.cell.id = c("BC10", "BC17"))

save(seurat_meta,file="seurat_meta.Rdata")

seurat_rec <- merge(x = sce11,

y = sce20,

add.cell.id = c("BC11", "BC20"))

save(seurat_rec,file="seurat_rec.Rdata")

memory.limit(40000)

library(Seurat)

library(dplyr)

seurat_primary[['percent.mt']]=PercentageFeatureSet(seurat_primary,pattern = "MT-")

VlnPlot(seurat_primary,features = c('nFeature_RNA','nCount_RNA','percent.mt'),ncol = 3)

seurat_primary <- subset(seurat_primary, subset = nFeature_RNA > 200

& nFeature_RNA < 5000&

percent.mt < 5)

seurat_primary <- NormalizeData(seurat_primary,

normalization.method = "LogNormalize",

scale.factor = 10000)

seurat_primary <- FindVariableFeatures(seurat_primary,

selection.method = "vst", nfeatures = 1000)

top10 <- head(VariableFeatures(seurat_primary), 10)

plot1 <- VariableFeaturePlot(seurat_primary)

plot2 <- LabelPoints(plot = plot1, points = top10, repel = TRUE)

plot1

plot2

all.genes <- rownames(seurat_primary)

seurat_primary <- ScaleData(seurat_primary,

features = all.genes)

seurat_primary <- RunPCA(seurat_primary, features = VariableFeatures(object = seurat_primary))

seurat_primary <- JackStraw(seurat_primary, num.replicate = 100)

seurat_primary <- ScoreJackStraw(seurat_primary, dims = 1:20)

JackStrawPlot(seurat_primary, dims = 1:20)

ElbowPlot(seurat_primary)

seurat_primary <- FindNeighbors(seurat_primary, dims = 1:20)

seurat_primary <- FindClusters(seurat_primary, resolution = 0.5)

head(Idents(seurat_primary))

seurat_primary <- RunUMAP(seurat_primary, dims = 1:20)

DimPlot(seurat_primary, reduction = "umap",label = F)

seurat_primary <- NormalizeData(seurat_primary,

normalization.method = "LogNormalize")

diff.wilcox = FindAllMarkers(seurat_primary)

head(diff.wilcox)

dim(diff.wilcox)

library(tidyverse)

all.markers = diff.wilcox %>% select(gene, everything()) %>%

subset(p_val_adj<0.05 & abs(diff.wilcox$avg_log2FC) > 0.5)

save(diff.wilcox,file = "diff_willcox.Rdata")

setwd("D:/WORK/bioinformatics/osteosarcoma/scRNA/primary")

save(all.markers,file = "all_markers.Rdata")

dim(all.markers)

summary(all.markers)

top10 <- all.markers %>% group_by(cluster) %>% top_n(n = 10, wt = avg_log2FC)

DoHeatmap(seurat_primary, features = top10$gene) + NoLegend()

library(SingleR)

#osteoblastic OS cells

VlnPlot(seurat_primary, features = c("COL1A1", "CDH11","RUNX2","IBSP"))

#chondroblastic OS cells

VlnPlot(seurat_primary, features = c("SOX9", "ACAN","PTH1R"))

#proliferating osteoblastic OS cells

VlnPlot(seurat_primary, features = c("TOP2", "MKI67","PCNA"))

#osteoclastic cells

VlnPlot(seurat_primary, features = c("ACP5", "CTSK","MMP9"))

#T cell

VlnPlot(seurat_primary, features = c("CD3D", "IL7R","CD8A","CD4","NKG7"))

#NK cell

VlnPlot(seurat_primary, features = c("GNLY", "NKG7"))

#Myeloid cells

VlnPlot(seurat_primary, features = c("CD74", "CD14","FCGR3A"))

#Fibroblasts

VlnPlot(seurat_primary, features = c("COL1A1", "LUM","DCN","COL3A1"))

#Mesenchymal stem cells

VlnPlot(seurat_primary, features = c("CXCL12", "SFRP2","MME","THY1"))

#Tregs

VlnPlot(seurat_primary, features = c("FOXP3", "IL2RA"))

#myoblasts

VlnPlot(seurat_primary, features = c("MYLPF", "MYL1"))

#endothelial cells

VlnPlot(seurat_primary, features = c("PECAM1", "VWF"))

#B cells

VlnPlot(seurat_primary, features = c("MS4A1", "CD19","JCHAIN"))

#M2 macrophages

VlnPlot(seurat_primary, features = c("CD163", "CD68","CD206"))

seurat_primary@meta.data$celltype[seurat_primary@meta.data$seurat_clusters==0]=c("Chondroblastic OS cells")

seurat_primary@meta.data$celltype[seurat_primary@meta.data$seurat_clusters==1]=c("Fibroblasts")

seurat_primary@meta.data$celltype[seurat_primary@meta.data$seurat_clusters==2]=c("T cells")

seurat_primary@meta.data$celltype[seurat_primary@meta.data$seurat_clusters==3]=c("Chondroblastic OS cells")

seurat_primary@meta.data$celltype[seurat_primary@meta.data$seurat_clusters==4]=c("Myeloid cells")

seurat_primary@meta.data$celltype[seurat_primary@meta.data$seurat_clusters==5]=c("Myeloid cells")

seurat_primary@meta.data$celltype[seurat_primary@meta.data$seurat_clusters==6]=c("M2 macrophages")

seurat_primary@meta.data$celltype[seurat_primary@meta.data$seurat_clusters==7]=c("Osteoblastic OS cells")

seurat_primary@meta.data$celltype[seurat_primary@meta.data$seurat_clusters==8]=c("Osteoblastic OS cells")

seurat_primary@meta.data$celltype[seurat_primary@meta.data$seurat_clusters==9]=c("Endothelial cells")

seurat_primary@meta.data$celltype[seurat_primary@meta.data$seurat_clusters==10]=c("Proliferating osteoblastic OS cells")

seurat_primary@meta.data$celltype[seurat_primary@meta.data$seurat_clusters==11]=c("Fibroblasts")

seurat_primary@meta.data$celltype[seurat_primary@meta.data$seurat_clusters==12]=c("NK cells")

seurat_primary@meta.data$celltype[seurat_primary@meta.data$seurat_clusters==13]=c("Novel1")

seurat_primary@meta.data$celltype[seurat_primary@meta.data$seurat_clusters==14]=c("Novel2")

seurat_primary@meta.data$celltype[seurat_primary@meta.data$seurat_clusters==15]=c("myoblasts")

DimPlot(seurat_primary, group.by="celltype",reduction = "umap",label = F)

FeaturePlot(

seurat_primary,

features = gene,pt.size = 0.5

)

FeaturePlot(

seurat_primary,

features = c(

"ATF4", "HILPDA","ATM","CBS","MUC1", "MT1G","PML","ARNTL"

),pt.size = 0.5

)

seurat_primary@assays$RNA@scale.data

exp = seurat_primary[["RNA"]]@counts

dim(exp)

exp[1:5,60:70]

resk_exp=exp[rownames(multicox_res),]

risk_exp=t(resk_exp)

fp=predict(res.cox1,risk_exp,type="risk")

fp=as.data.frame(fp)

setwd("D:/WORK/bioinformatics/osteosarcoma/scRNA/primary")

save(seurat_primary,file="seurat_primary.Rdata")

genelist=c("ATF4", "HILPDA","ATM","CBS","MUC1", "MT1G","PML","ARNTL")

save(genelist,file="sig_gene.Rdata")

fp$risk_group[fp$fp>median(fp$fp)]="High_risk"

fp$risk_group[fp$fp<median(fp$fp)]="Low_risk"

fp=fp[rownames(seurat_primary@meta.data),]

seurat_primary@meta.data$risk_group=fp$risk_group

DimPlot(seurat_primary, group.by="risk_group",reduction = "umap",pt.size=0.5,label = F)

save(seurat_primary,file = "meta_fp.Rdata")

save(res.cox1,multicox_res,file="risk_judge.Rdata")

#####

#####m

library(monocle)

library(dplyr)

data <- as(as.matrix(seurat_primary@assays$RNA@counts), 'sparseMatrix')

pd <- new('AnnotatedDataFrame', data = seurat_primary@meta.data)

fData <- data.frame(gene_short_name = row.names(data), row.names = row.names(data))

fd <- new('AnnotatedDataFrame', data = fData)

mycds <- newCellDataSet(data,

phenoData = pd,

featureData = fd,

expressionFamily = negbinomial.size())

setwd("D:/WORK/bioinformatics/osteosarcoma/scRNA/primary")

save(mycds, file = "mycds_raw.Rdata")

rm(list = ls())

load("mycds_raw.Rdata")

library("monocle")

mycds <- estimateSizeFactors(mycds)

mycds <- estimateDispersions(mycds)

load("../../tmp/markergene.Rdata")

markers.gene <- all.markers$gene

mycds <- setOrderingFilter(mycds, markers.gene)

mycds <- reduceDimension(mycds, max_components = 2, method = 'DDRTree')

mycds <- orderCells(mycds)

save(mycds,file = "../../tmp/mycds_reduced.Rdata")

p1 <- plot_cell_trajectory(mycds, color_by = "seurat_clusters")

ggsave("../../out/3.4trajectory_1.pdf", plot = p1)

p2 <- plot_cell_trajectory(mycds, color_by = "State")

ggsave("../../out/3.4trajectory_2.pdf", plot = p2)

rm(list = ls())

library(Seurat)

library(tidyverse)

library(patchwork)

library(monocle)

library(clusterProfiler)

library(org.Hs.eg.db)

dge.State <- FindMarkers(seurat_primary, ident.1 = "High_risk", ident.2 = "Low_risk", group.by = 'risk_group')

sig_dge.High <- subset(dge.State, p_val<0.05&avg_log2FC>0)

sig_dge.Low <- subset(dge.State, p_val<0.05&avg_log2FC< 0)

ego_MF <- enrichGO(gene = row.names(sig_dge.High),

#universe = row.names(dge.celltype),

OrgDb = 'org.Hs.eg.db',

keyType = 'SYMBOL',

ont = "MF",

pAdjustMethod = "BH",

pvalueCutoff = 0.01,

qvalueCutoff = 0.05)

ego_BP <- enrichGO(gene = row.names(sig_dge.High),

#universe = row.names(dge.celltype),

OrgDb = 'org.Hs.eg.db',

keyType = 'SYMBOL',

ont = "BP",

pAdjustMethod = "BH",

pvalueCutoff = 0.01,

qvalueCutoff = 0.05)

p_BP <- barplot(ego_BP,showCategory = 10) + ggtitle("barplot for Biological process")

p_MF <- barplot(ego_MF,showCategory = 10) + ggtitle("barplot for Molecular function")

plotc <- p_BP/p_MF

genelist1 <- bitr(row.names(sig_dge.High), fromType="SYMBOL",

toType="ENTREZID", OrgDb='org.Hs.eg.db')

genelist1 <- pull(genelist1,ENTREZID)

ekegg1 <- enrichKEGG(gene = genelist1, organism = 'hsa')

barplot(ekegg1, showCategory=20)

genelist2 <- bitr(row.names(sig_dge.Low), fromType="SYMBOL",

toType="ENTREZID", OrgDb='org.Hs.eg.db')

genelist2 <- pull(genelist2,ENTREZID)

ekegg2 <- enrichKEGG(gene = genelist2, organism = 'hsa')

barplot(ekegg2, showCategory=17)

setwd("D:/WORK/bioinformatics/osteosarcoma/scRNA/primary")

highrisk_gene=rownames(sig_dge.High)

highrisk_gene=as.data.frame(highrisk_gene)

lowrisk_gene=rownames(sig_dge.Low)

lowrisk_gene=as.data.frame(lowrisk_gene)

setwd("D:/WORK/bioinformatics/osteosarcoma/scRNA/primary")

write.csv(highrisk_gene,file = "highrisk_gene.csv")

write.csv(lowrisk_gene,file = "lowrisk_gene.csv")

highrisk_kegg=read.csv("highrisk_kegg.csv")

lowrisk_kegg=read.csv("lowrisk_kegg.csv")

library(ggplot2)

mytheme=theme(plot.title = element_text(face = "bold",size = "12",hjust = 0.5),

axis.title = element_text(face = "bold",size = "10"),

axis.text = element_text(size = "9"),

axis.line = element_line(size = 1),

axis.text.x = element_text(angle = 0,vjust =0.7),

#panel.grid.major.y = element_line(color = "grey",linetype = 1),

#panel.grid.minor.y = element_line(color = "grey",linetype = 2),

#panel.grid.major.x = element_line(color = "grey",linetype = 1),

#panel.grid.minor.x = element_line(color = "grey",linetype = 2),

panel.background = element_rect(fill = "white",color = "black",size=1.5),

panel.grid.major = element_blank())

library(RColorBrewer)

library(stringr)

fix(highrisk_kegg)

highrisk_kegg$pvalue=as.numeric(highrisk_kegg$pvalue)

highrisk_kegg$Count=as.numeric(highrisk_kegg$Count)

highrisk_kegg$pvalue=factor(highrisk_kegg$pvalue,order=TRUE,

levels =highrisk_kegg$pvalue)

ggplot(data=highrisk_kegg)+geom_bar(mapping=aes(

x=Count,y=Description,fill=pvalue),stat="identity") +

mytheme+scale_fill_gradient(low = "red", high = "blue")

lowrisk_kegg$pvalue=as.numeric(lowrisk_kegg$pvalue)

lowrisk_kegg$Count=as.numeric(lowrisk_kegg$Count)

lowrisk_kegg$pvalue=factor(lowrisk_kegg$pvalue,order=TRUE,

levels =lowrisk_kegg$pvalue)

ggplot(data=lowrisk_kegg)+geom_bar(mapping=aes(

x=Count,y=Description,fill=pvalue),stat="identity") +

mytheme+scale_fill_gradient(low = "red", high = "blue")

genelist=c("ATF4", "HILPDA","ATM","CBS","MUC1", "MT1G","PML","ARNTL")

a=seurat_rec[genelist,]

counts<-seurat_rec@assays$RNA@data

data<-data.frame(counts[genelist,])

celltype=as.data.frame(seurat_rec@meta.data$orig.ident)

data=t(data)

data=as.data.frame(data)

data$celltype=celltype$'seurat_rec@meta.data$orig.ident'

setwd("D:/WORK/bioinformatics/osteosarcoma/scRNA/primary")

save(dat,data,file="genelistdata.Rdata")

data$group <- ifelse(data$ATF4>0,"positive","negative")

ATF4 <- select(data,celltype,group)

ATF4 <- na.omit(ATF4)

names(ATF4)[2] <- "ATF4"

data$group <- ifelse(data$HILPDA>0,"positive","negative")

HILPDA <- select(data,celltype,group)

HILPDA <- na.omit(HILPDA)

names(HILPDA)[2] <- "HILPDA"

data$group <- ifelse(data$ATM>0,"positive","negative")

ATM <- select(data,celltype,group)

ATM <- na.omit(ATM)

names(ATM)[2] <- "ATM"

data$group <- ifelse(data$CBS>0,"positive","negative")

CBS <- select(data,celltype,group)

CBS <- na.omit(CBS)

names(CBS)[2] <- "CBS"

data$group <- ifelse(data$MUC1>0,"positive","negative")

MUC1 <- select(data,celltype,group)

MUC1 <- na.omit(MUC1)

names(MUC1)[2] <- "MUC1"

data$group <- ifelse(data$MT1G>0,"positive","negative")

MT1G <- select(data,celltype,group)

MT1G <- na.omit(MT1G)

names(MT1G)[2] <- "MT1G"

data$group <- ifelse(data$PML>0,"positive","negative")

PML <- select(data,celltype,group)

PML <- na.omit(PML)

names(PML)[2] <- "PML"

data$group <- ifelse(data$ARNTL>0,"positive","negative")

ARNTL <- select(data,celltype,group)

ARNTL <- na.omit(ARNTL)

names(ARNTL)[2] <- "ARNTL"

library(ggplot2)

chisq.test(table(ATF4$celltype,ATF4$ATF4))

ATF4$celltype <- "cells"

mytheme=theme(plot.title = element_text(face = "bold",size = "12",hjust = 0.5),

axis.title = element_text(face = "bold",size = "10"),

axis.text = element_text(size = "9",colour = "black"),

axis.line = element_line(size = 1),

#axis.text.x = element_text(angle = 45,vjust =0.7),

panel.grid.major.y = element_line(color = "grey",linetype = 3),

panel.grid.minor.y = element_line(color = "grey",linetype = 2),

panel.grid.major.x = element_line(color = "grey",linetype = 3),

panel.grid.minor.x = element_line(color = "grey",linetype = 2),

panel.background = element_rect(fill = "white",color = "black",size=1.5),

panel.grid.major = element_blank())

ggplot(ATF4, aes(celltype)) + geom_bar(aes(fill=ATF4), position="fill")+ylab("Propotion")+

mytheme

chisq.test(table(HILPDA$celltype,HILPDA$HILPDA))

HILPDA$celltype <- "cells"

ggplot(HILPDA, aes(celltype)) + geom_bar(aes(fill=HILPDA), position="fill")+ylab("Propotion")+

mytheme

chisq.test(table(ATM$celltype,ATM$ATM))

ATM$celltype <- "cells"

ggplot(ATM, aes(celltype)) + geom_bar(aes(fill=ATM), position="fill")+ylab("Propotion")+

mytheme

chisq.test(table(CBS$celltype,CBS$CBS))

CBS$celltype <- "cells"

ggplot(CBS, aes(celltype)) + geom_bar(aes(fill=CBS), position="fill")+ylab("Propotion")+

mytheme

chisq.test(table(MUC1$celltype,MUC1$MUC1))

MUC1$celltype <- "cells"

ggplot(MUC1, aes(celltype)) + geom_bar(aes(fill=MUC1), position="fill")+ylab("Propotion")+

mytheme

chisq.test(table(MT1G$celltype,MT1G$MT1G))

MT1G$celltype <- "cells"

ggplot(MT1G, aes(celltype)) + geom_bar(aes(fill=MT1G), position="fill")+ylab("Propotion")+

mytheme

chisq.test(table(PML$celltype,PML$PML))

PML$celltype <- "cells"

ggplot(PML, aes(celltype)) + geom_bar(aes(fill=PML), position="fill")+ylab("Propotion")+

mytheme

chisq.test(table(ARNTL$celltype,ARNTL$ARNTL))

ARNTL$celltype <- "cells"

ggplot(ARNTL, aes(celltype)) + geom_bar(aes(fill=ARNTL), position="fill")+ylab("Propotion")+

mytheme

####

####Drugs

setwd("D:/WORK/bioinformatics/osteosarcoma/OS_Ferroptosis/TARGET/clusters/NMF")

load("cell_expression.rdata")

library(data.table)

phe=fread("GPL13667-15572.txt",header = T)

phe=phe[,c(1,15)]

phe=phe[phe$ID %in% rownames(combat_eset),]

phe=as.data.frame(phe)

dim(combat_eset)

rownames(phe)=phe$ID

phe=phe[rownames(combat_eset),]

all(rownames(phe)==rownames(combat_eset))

phe$median=apply(combat_eset,1,median)

phe=phe[order(phe$`Gene Symbol`,phe$median,decreasing = T),]

phe=phe[!duplicated(phe$`Gene Symbol`),]

combat_eset=combat_eset[phe$ID,]

rownames(combat_eset)=phe$`Gene Symbol`

ids=phe

phe=fread("cell.txt")

phe=phe[,c(3,17)]

phe=phe[phe$`Array Data File`%in% colnames(combat_eset),]

combat_eset=combat_eset[,phe$`Array Data File`]

all(colnames(combat_eset)==phe$`Array Data File`)

colnames(combat_eset)=phe$`Characteristics[cell line]`

setwd("D:/WORK/bioinformatics/osteosarcoma/OS_Ferroptosis/TARGET/clusters/NMF/Rdata")

save(combat_eset,file="drugexpression.rdata")

library(stringr)

rownames(combat_eset)=str_split(ids$`Gene Symbol`," /// ",simplify = T)[,1]

load("drugexpression.rdata")

geneset=rownames(combat_eset)

geneset=as.data.frame(geneset)

multicox_res$gene.symbol[multicox_res$gene.symbol=="HILPDA"]="C7orf68"

risk_exp=combat_eset[multicox_res$gene.symbol,]

risk_exp=t(risk_exp)

colnames(risk_exp)[colnames(risk_exp)=="C7orf68"]="HILPDA"

risk_exp=as.data.frame(risk_exp)

fp=predict(res.cox1,risk_exp,type="risk")

fp=as.data.frame(fp)

score=fp

save(score,file = "drugscore.Rdata")

rm(list=ls())

options(stringsAsFactors = F)

load("drugscore.rdata")

score=as.data.frame(score)

score$cell=rownames(score)

setwd("D:/WORK/bioinformatics/osteosarcoma/OS_Ferroptosis/TARGET/clusters/NMF")

drug2=read.csv("GDSC2_fitted_dose_response_25Feb20.csv",header=T)

drug1=read.csv("GDSC1_fitted_dose_response_25Feb20(1).csv",header = T)

drug2=rbind(drug1,drug2)

score=score[rownames(score)%in% unique(drug2$CELL_LINE_NAME),]

score=as.data.frame(score)

drug3=drug2[drug2$CELL_LINE_NAME%in% score$cell,]

drug3=drug3[order(drug3$CELL_LINE_NAME),]

a=as.data.frame(table(drug3$CELL_LINE_NAME))

score=score[order(score$cell),]

score=as.data.frame(score)

score1=unlist(lapply(1:235, function(x) rep(score$fp[x],a$Freq[x])))

drug3$score=score1

drug3=drug3[order(drug3$DRUG_NAME),]

b=as.data.frame(table(drug3$DRUG_NAME))

corx=function(x){

cor1=drug3[drug3$DRUG_NAME==b$Var1[x],]

cor1=cor1[,c(16,20)]

cor2=cor(cor1,method = "spearman")

cor3=cor2[1,2]

names(cor3)=b$Var1[x]

return(cor3)

}

library(corrplot)

corp=function(x){

cor1=drug3[drug3$DRUG_NAME==b$Var1[x],]

cor1=cor1[,c(16,20)]

re1=cor.mtest(cor1,conf.level=0.95)

re1=re1$p

cor4=re1[1,2]

names(cor4)=b$Var1[x]

return(cor4)

}

c=lapply(1:449, function(x) corx(x))

c=unlist(c)

c=as.data.frame(c)

colnames(c)="cor"

cp=lapply(1:449, function(x) corp(x))

cp=unlist(cp)

cp=as.data.frame(cp)

colnames(cp)="p_value"

c$order=rep("cc",449)

colnames(drug2)

drug2=drug2[,c(9,11)]

colnames(drug2)=c("drug","pathway")

drug2=drug2[!duplicated(drug2$drug),]

c=c[drug2$drug,]

c$pathway=drug2$pathway

all(rownames(c)==rownames(cp))

c=c[rownames(cp),]

c$p_value=cp[,1]

c2=c[abs(c$cor)>0.15&c$p_value<0.05,]

c2=c2[order(c2$cor,decreasing = T),]

c2$pvalue=-log10(c2$p_value)

c2$name=rownames(c2)

library(ggplot2)

mytheme=theme(plot.title = element_text(face = "bold",size = "12",hjust = 0.5),

axis.title = element_text(face = "bold",size = "10"),

axis.text = element_text(size = "9"),

axis.line = element_line(size = 1),

axis.text.x = element_text(angle = 45,vjust =0.7),

#panel.grid.major.y = element_line(color = "grey",linetype = 1),

#panel.grid.minor.y = element_line(color = "grey",linetype = 2),

#panel.grid.major.x = element_line(color = "grey",linetype = 1),

#panel.grid.minor.x = element_line(color = "grey",linetype = 2),

panel.background = element_rect(fill = "white",color = "black",size=1.5),

panel.grid.major = element_blank())

library(RColorBrewer)

display.brewer.all()

c2=c2[order(c2$cor,decreasing = F),]

c2$name=factor(c2$name,order=TRUE,levels =c2$name)

ggplot(data=c2)+geom_bar(mapping=aes(x=name,y=cor,fill=pvalue),stat="identity") +

mytheme+scale_fill_gradient(low = "red", high = "purple")

c=c2

c$group=ifelse(c$cor>0,"drug_ressistance","drug_sensitivity")

c$cors=abs(c$cor)

c1=c[c$pathway%in%c("PI3K/MTOR signaling","DNA replication","ERK MAPK signaling"," RTK signaling",

"Genome integrity","Other","Mitosis","EGFR signaling","Chromatin histone acetylation","Cell cycle",

"Apoptosis regulation"),]

c2=c1[c1$pathway %in% c("Apoptosis regulation","Cell cycle","Chromatin histone acetylation","Metabolism",

"EGFR signaling","RTK signaling","Cytoskeleton","PI3K/MTOR signaling","WNT signaling"),]

c2$pathway=factor(c2$pathway,order=TRUE,levels =c("Apoptosis regulation","Cell cycle","Chromatin histone acetylation","Metabolism",

"EGFR signaling","RTK signaling","Cytoskeleton","PI3K/MTOR signaling","WNT signaling"))

ggplot(data=c2)+geom_point(mapping=aes(x=name,y=pathway,color=cor,size=pvalue)) + scale_color_gradient(low="blue",high = "red")+mytheme

ggplot(data=c)+geom_bar(mapping = aes(x=pathway,fill=group))+mytheme+coord_flip()
